# Survival Analysis of Multi-Omics Data Identifies Potential Prognostic Markers of Pancreatic Ductal Adenocarcinoma

**DOI:** 10.3389/fgene.2019.00624

**Published:** 2019-07-18

**Authors:** Nitish Kumar Mishra, Siddesh Southekal, Chittibabu Guda

**Affiliations:** Department of Genetics, Cell Biology and Anatomy, University of Nebraska Medical Center, Omaha, NE, United States

**Keywords:** Dm-CpG: Differentially methylated CpG, DMR: differentially methylated region, DEG: differentially expressed gene, HR: hazard ratio, TCGA: The Cancer Genome Atlas, GDC: The Genomic Data Commons, FDR: false discovery rate

## Abstract

Pancreatic ductal adenocarcinoma (PDAC) is the most common and among the deadliest of pancreatic cancers. Its 5-year survival is only ∼8%. Pancreatic cancers are a heterogeneous group of diseases, of which PDAC is particularly aggressive. Like many other cancers, PDAC also starts as a pre-invasive precursor lesion (known as pancreatic intraepithelial neoplasia, PanIN), which offers an opportunity for both early detection and early treatment. Even advanced PDAC can benefit from prognostic biomarkers. However, reliable biomarkers for early diagnosis or those for prognosis of therapy remain an unfulfilled goal for PDAC. In this study, we selected 153 PDAC patients from the TCGA database and used their clinical, DNA methylation, gene expression, and micro-RNA (miRNA) and long non-coding RNA (lncRNA) expression data for multi-omics analysis. Differential methylations at about 12,000 CpG sites were observed in PDAC tumor genomes, with about 61% of them hypermethylated, predominantly in the promoter regions and in CpG-islands. We correlated promoter methylation and gene expression for mRNAs and identified 17 genes that were previously recognized as PDAC biomarkers. Similarly, several genes (B3GNT3, DMBT1, DEPDC1B) and lncRNAs (PVT1, and GATA6-AS) are strongly correlated with survival, which have not been reported in PDAC before. Other genes such as EFR3B, whose biological roles are not well known in mammals are also found to strongly associated with survival. We further identified 406 promoter methylation target loci associated with patients survival, including known esophageal squamous cell carcinoma biomarkers, cg03234186 (ZNF154), and cg02587316, cg18630667, and cg05020604 (ZNF382). Overall, this is one of the first studies that identified survival associated genes using multi-omics data from PDAC patients.

## Introduction

Pancreatic ductal adenocarcinoma (PDAC) originates from the ductal epithelial cells of the pancreas and it is the most common malignancy of the pancreas. Due to lack of early symptoms, PDAC is commonly presented in the metastatic stage, and as a result, fewer than 20% patients can be considered for surgical removal of the tumors (Adamska et al., 2017). Unfortunately, removing frank tumors from the pancreas cannot be expected to cure a metastatic disease, which is reflected in the current statistics of 5-year survival, which remains pegged at a dismal 8% (Chiaravalli et al., 2017). By 2030, PDAC is projected to become the second leading cause of mortality from cancer, only behind lung cancer (Rahib et al., 2014). This is the most alarming situation, and we have an urgent need for developing early detection and effective treatment regimens.

Recent studies regarding molecular profiling and epigenetic regulation in PDAC pathophysiology have provided a valuable roadmap for this effort. We are beginning to gather information about the early-onset and PDAC-specific epigenetic alterations that alter gene expression (Neureiter et al., 2014), especially those that induce metastatic changes such as genome structure reorganization and affect tumor grade, stage, and patient survival (Thompson et al., 2015). Such studies are helping in identifying targets for designing epigenetic inhibitors to treat PDAC. Not surprisingly, these targets belong to growth signaling and tumor suppressor-silencing pathways, and also those that affect cell cycle checkpoints (Paradise et al., 2018).

There is also no doubt that early detection and early beginning of therapy will be key for defeating PDAC. Identification of early-onset DNA methylations in PDAC target genes should provide biomarker candidates for early diagnosis. We also know from earlier studies that certain critical genes are hypomethylated in pancreatic cancer. The mucin 4 (MUC4) gene is one example of promoter hypomethylation in pancreatic cancer (Zhu et al., 2011). However, pancreatic cancer appears to be affected by both hyper- and hypomethylated genes (Mishra and Guda, 2017). In particular, inside the promoters of ∼72% of human genes, there are stretches of CpG dinucleotides (known as CpG islands), which are hypermethylated in cancer (Saxonov et al., 2006). Frequently, transcription of tumor suppressor genes is silenced by CpG island hypermethylation, while hypomethylation of promoters appears to cause overexpression of oncogenes and genomic instability (Tan et al., 2009). Abnormal DNA methylation affects many genes of cancer patients. In PDAC, genes involved in axon guidance, cell adhesion, epithelial-mesenchymal transition (EMT), and other pathways of tumor development, as well as genes involved in pancreatic development including the HOX-family genes, show abnormal DNA methylation (Nones et al., 2014; Mishra and Guda, 2017). Some of these genes may be useful for diagnosing PDAC stage and for the prognosis of successful therapy.

The availability of bisulfite-sequencing and array-based DNA methylation data in The Cancer Genome Atlas (TCGA) (Weinstein et al., 2013; Tomczak et al., 2015), and International Cancer Genome Consortium (ICGC) (Zhang et al., 2011) has given our pursuit for identifying candidate biomarkers a great fillip. The study of differentially methylated loci between tumor and normal samples has great scientific merit for cataloging the genomic changes in PDAC. But integrated genomic analysis of differences in DNA methylations, their impact on expression of the genes, and correlating those data with patient survival will bring us closer to the goal of identifying the candidate biomarkers. Until recently, integrative analyses have mostly been done for examining methylation status of promoters and CpG islands (Vincent et al., 2011). For example, Raphael et al. used integrative analysis of TCGA pancreatic ductal cancer data (Raphael et al., 2017), but their focus was somatic alterations and molecular subtyping. Using the TCGA data, a number of DNA methylation pattern analyses have been reported for multiple cancers (Noushmehr et al., 2010; Aine et al., 2015; Yang et al., 2015); but for PDAC, this is still lacking. Unlike in our previous study (Mishra and Guda, 2017), in which we performed integrative analysis of all types of pancreatic cancers (PC) in the TCGA database, the present work is focused exclusively on PDAC, that is, this report does not contain any other subtypes of PC. In this PDAC study, we analyzed differential DNA methylation, gene expression, miRNA and lncRNA expression, and association of promoter DNA methylation with gene expression and lncRNA expression (**Figure S1**). Next, we examined whether those genomic and transcriptional changes corresponded with patient survival in a significant way. Overall, in the current study, we identified several prognostic markers for pancreatic ductal cancer.

## Materials and Methods

### Clinical Data and Samples

We downloaded the current study view clinical data as of August 2018 from cBioPortal (Gao et al., 2013). The TCGA database has a total of 186 pancreatic cancer patients. Based on the described neoplastic and histological information of these patients in the clinical files, we selected 154 patients who had PDAC unambiguously. We excluded the other patients who had endocrine, invasive adenocarcinoma, undifferentiated or mixed pancreatic cancers (**Table S2**). CpGs/genes/miRNAs/lncRNAs with missing values in ≥20% samples, and similarly, samples with missing values of ≥20% of CpGs/genes/miRNAs/lncRNAs were excluded from further analysis.

### DNA Methylation, RNAseq, and miRNAseq Data

The Bioconductor tool *TCGAbiolinks* (Colaprico et al., 2016) was used to download the TCGA level-3 data on DNA methylation (Illumina HumanMethylation450 BeadArray), gene expression (IlluminaHiSeq RNASeqV2), and lncRNA and microRNA expression (IlluminaHiSeq miRNAseq). The DNA methylation data also contains β values for 485,577 CpG sites with annotations for transcripts from GENCODE v22, the associated CpG island (CGI), CpG sites’ distance from the nearest transcription start site (TSS), and CpG coordinates as per GRCh38 reference genome. The β values are calculated as (M/M+U) which ranges between 0 and 1, where M is the methylated allele frequency and U is the unmethylated allele frequency. Therefore, a higher β values indicate a higher level of methylation. The gene expression data were obtained for each of the 60,483 GENCODE v22 genes in each sample. The miRNASeq data for each sample have single raw read counts and reads-per-million (RPM) counts for 1,881 miRNAs that are annotated in miRBase v21. As TCGA PDAC samples were processed in batches at different sites of the consortium, the data can be vulnerable to batch effects. Before starting the PDAC data analysis we first checked for possible batch effect in different types of data using Mbatch (Akbani et al., 2010).

### Methylation Data Processing

Beta values of CpG probes mapped against X, Y, and mitochondrial chromosomes were excluded from analyses to eliminate gender bias. CpGs with missing β values (approximately 20% of the samples) were also excluded. To estimate the remaining missing values in the data, we used the k-nearest neighbor-based imputation method using the *imputeKNN* module of the R tool (R Core Team, 2019), *impute* (Troyanskaya et al., 2001). We also removed the data from CpG probes which overlapped with repeat masker and SNPs from dbSNP v151 with minor allele frequency (MAF) > 1% (Zhou et al., 2017). Statistical analyses of DNA methylation of 162 samples (153 primary tumors and nine normal samples) were performed at two different levels, i.e., the CpG site level, and the region level.

CpG probes were independently mapped in six different subregions of the genes: TSS200 (the region from TSS to 200 bp upstream of TSS), TSS1500 (200–1,500 bp upstream from TSS), 5’UTR, 1st exon, gene body, and 3’UTR. DNA methylation characteristics in the known UCSC CpG island, shores (regions 0–2 kb from CpG islands), and shelfs (regions 2–4 kb from CpG islands) were also analyzed.

### Logistic Regression Analysis

We used logistic regression in R to classify the tumor and normal samples on the basis of their DNA methylation, gene expression, lncRNA expression, and miRNA expression data. Logistic regression was performed by using *lm* function in R. R package, *ROCR* was used to evaluate logistic regression performance, calculate the area under curve (AUC), and generate receiver operating characteristic (ROC) curve plots (Sing et al., 2005).

### Differential Methylation Analysis

The β values for CpGs after preprocessing and imputation analyses were further normalized by using the beta mixed integer-quantile normalization (BMIQ) tool to adjust for type I and type II probes in data by using R tool, *BMIQ* (Teschendorff et al., 2013). The R package, *limma* was used for conducting supervised differential methylation analyses. For a CpG site to be considered differentially methylated, the primary tumor and normal samples were to have a mean β value difference of at least 0.2 (∆β ≥ 0.2), and the BH adjusted *p*-value less than 0.005. Using the R tool, *gtrellis*, we generated circular plots of 10 Mb sliding windows for each chromosome to examine differentially methylated CpGs that had differential methylation frequencies (Gu et al., 2016). Next, we determined the methylation frequency per megabase pair (Mb) for each chromosome by calculating the total number of dm-CpGs in the chromosome and dividing by the length of the chromosome (Mb) using the GRCh38. Hypermethylation and hypomethylation frequencies were also calculated for each autosomal chromosome in a similar manner. For each chromosome, when the ratio between hypermethylation to hypomethylation frequencies was ≥1.5, we considered that chromosome to be predominantly hypermethylated. On the other hand, if the hypomethylation to hypermethylation frequency ratio is ≥1.5 we considered that chromosome to be predominately hypomethylated.

### Differentially Methylated Regions (DMRs) Analysis

Differentially methylated region (DMR) analyses were performed using the Bioconductor tool *DMRcate* (Peters et al., 2015). *DMRcate* first calculates differential methylation at individual CpG sites derived by using moderated *t*-statistic from *limma* (Ritchie et al., 2015). After correcting for false discovery rate (FDR), regions of significant dm-CpGs were agglomerated into groups where the distance between two consecutive probes is within 1 kb. Only those DMRs that have at least two dm-CpGs with adjusted *p*-value < 0.01 within 1-kb distance were considered for DMR analysis. Next, we annotated the overlapping promoter regions (+/−2,000 bp from TSS) and generated a plot of DMRs by using the Bioconductor package *Gviz*.

### RNASeq and miRNASeq Data Processing

The TCGA level-3 RNASeq data contain a single raw read count and a normalized expression value for each gene. In contrast, the GDC data portal has different types of level-3 data. From the GDC, we used HT-Seq raw read counts data for differential gene expression and the FPKM-UQ for correlation analysis. These expression values were generated by aligning the reads with the GRCh38 reference genome and then quantifying the mapped reads for the genes. TCGA level-3 miRNASeq data contain raw read count for each miRNA in the miRBase database, which was derived by exact mapping of miRNASeq data (Chu et al., 2016).

### Differential Gene Expression Analysis

For differential gene expression analysis, the expected counts data from 146 primary PDAC and three normal samples were used. Before differential expression analysis, we removed all genes with missing expression values (∼20% of the samples) and also genes which had CPM (count per million) numbers less than one (about 25% of the samples). After preprocessing, we used the Bioconductor tool, *DESeq2* (Love et al., 2014) for differential gene expression analysis, for which, a cutoff value of 0.01 for both raw *p*-value and Benjamini–Hochberg (BH) (Benjamini and Hochberg, 1995) adjusted *p*-value were applied. For differential miRNA analysis, we used raw read counts in *DESeq2* with a BH adjusted *P*-value of ≤0.01.

### Correlation Between DNA Methylation and Gene Expression

For the correlation analysis, primary tumor samples of 146 patients that contained both DNA methylation and gene expression data were used. Correlation between promoter DNA methylation and corresponding gene expression was done by using linear regression function in the R package, *cor.test*. Methylation and expression levels (log2 (FPKM-UQ + 1) of genes were tested for non-zero correlation using Spearman’s correlation, after excluding all samples with a correlation value of zero. Any association between DNA methylation and gene expression was considered as significant if the *p*-value ≤ 0.005 and rho ≥|0.25|.

### Pathway Enrichment Analysis

Bioconductor package, *clusterProfiler* (Yu et al., 2012) was used for enrichment analysis of differentially expressed genes (DEG). KEGG canonical pathways were used for pathway enrichment analysis. We used BH adjustment *p*-values of 0.05 and a minimum of five and maximum of 500 genes as selection criteria for every significant pathway. For the pathway enrichment analysis of dm-CpGs, we used ‘*gometh*’ module of Bioconductor tool *missMethyl* (Phipson et al., 2016). Genes associated with dmCpGs (Δβ ≥ 0.2) in the Illumina Human 450K BeadChip are obtained from the annotation package, *IlluminaHumanMethylation450kanno.ilmn12.hg19*. All GO and KEGG terms were tested using ‘*gometh*’ function, and false discovery rates were calculated using the BH method.

### Survival Analysis

To reveal the roles of differentially expressed genes and miRNAs on patient survival, PDAC patients were classified into high and low expression groups, using the median expression of genes as the cut-off value. For the analysis of promoter region DNA methylations, we used β value cutoff of ≥0.5 (high) and ≤0.3 (low) groups. We analyzed only those CpG sites that were differentially methylated (±1,500 bp from TSS) and also negatively correlated with gene expression. We used the R tool, *survival*, for survival analysis, and Kaplan–Meier (KM) survival plots were generated. In addition, we performed Cox-regression analyses. For both analyses, we selected CpGs that had *p*-value ≤ 0.05. For gene expression, miRNA, and lncRNA expression and patient survival analyses, we used all available genes in the analysis and divided PDAC patients into two classes based on the median expression. PDAC patients that were above the median, were classed as the high expression group, and those below the median were classed as the low expression group.

## Results

We downloaded level-3 DNA methylation, gene expression, and miRNA expression data from TCGA using Bioconductor tool, *TCGAbiolinks*, and systematically carried out data cleaning, global unsupervised analyses, and detailed individual and integrative analyses on DNA methylation, mRNA, and miRNA expression datasets. To understand the functional significance and relevance of the differentially-expressed and differentially-methylated genes in PDAC, we also performed downstream analyses using pathway enrichment tools and Cox-regression and Kaplan–Meier survival plots. Complete flow-chart of the data analysis is available in **Figure S1**.

### Global DNA Methylation Analysis

We performed the Wilcoxon rank test to analyze the overall difference in DNA methylation levels in six different gene sub-regions (TSS200, TSS1500, 1st exon, 5´UTR, 3´UTR, and gene-body) and five methylated genomic regions (CpG-island, s-shore, n-sore, s-shelf, and n-shelf). For this analysis, we combined the β values of all CpGs in corresponding regions for tumor and normal samples. Our analyses revealed that CpG segments close to TSS and also the islands themselves have, in general, a higher level of DNA methylation in tumor samples (**Figure S2**). Specifically, DNA methylation levels of TSS200, TSS1500, 1st exon, 5´UTR, island, s-shore, and n-shore regions were higher in the tumor. In contrast, DNA methylation levels were low in genomic regions that are away from the TSS and the CpG islands (**Figure S2**).

**Figure 1:**
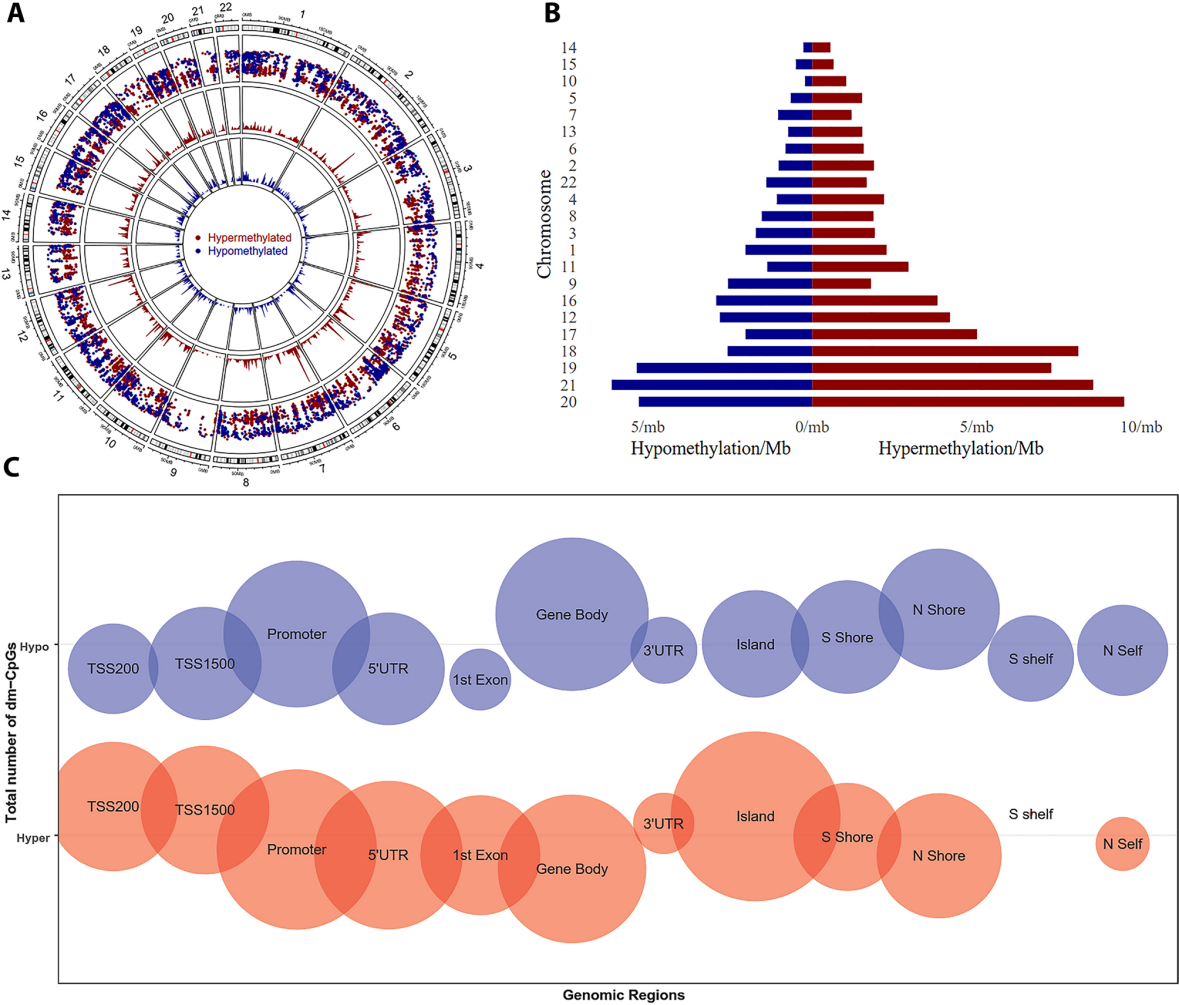
Differential DNA methylation distribution. **(A)** Circular plot of CpGs, chromosomes are shown in a clockwise direction from 1 to 22 in the outermost circle. Chromosomes X, Y, and M were excluded from analysis. The two innermost circles represent the differential hypermethylation and hypomethylation frequencies in a 10 Mb sliding window across the genome. **(B)** Pyramid (stacked) plot for differential hyper and hypomethylation frequencies for each chromosome. Chromosomes are sorted based on total differential methylation in per megabase pair length of the chromosomes. **(C)** Bubble plot of differentially methylated CpGs in genomic regions. Size of bubble represents a total number of dm-CpGs.

We observed a total 12,083 differentially methylated CpGs (dm-CpGs) with ∆β ≥ |0.2| between tumor and normal samples; out of these 7,378 were hypermethylated and 4,705 were hypomethylated (**Table S3**, **Figure S3**). At even higher thresholds (∆β ≥ |0.3|), the number of dm-CpG sites dwindled to 1,741. **Figure 1A** shows all dm-CpG results from each autosomal chromosome at ∆β ≥|0.2| depicted in the outer circle of the circos plot. The two innermost circles show the density of hyper- and hypomethylation in a 10 Mb sliding window across the genome. The distribution of dm-CpGs in twelve different genomic subregions is shown in **Table 1** and **Figure 1C**. A total 4,610 dm-CpGs were observed within the promoter regions of genes i.e., ±1.5 Kb from the TSS of genes. We also observed that the regions close to the CpG islands (island, shore) and the promoters (TSS200, TSS1500, promoter, 1st Exon, 5´UTR), were predominantly hypermethylated (**Figure S2**—1.5kb distribution plot), while regions away from promoter (shelf) and promoter (3´UTR, gene body) are hypomethylated (**Table 1**, **Figure 1C**).

**Table 1:** Distribution of differentially methylated CpG sites in different genomic and gene regions in pancreatic ductal adenocarcinoma (∆β ≥ 0.2).

Genomic region	dm-CpG	Hypermethylated	Hypomethylated
3UTR	310	165	145
5UTR	1,144	1,935	433
1^st^ Exon	815	682	133
Body	3,815	1,935	1,880
TSS200	1,172	935	237
TSS1500	1,536	915	441
Island	5,241	4,870	371
N Shore	1,378	807	571
N Self	388	148	240
S Shore	916	472	444
S shelf	320	105	215
Promoter	4,610	3,174	1,436

In PDAC tumors, we observed that chromosome 1 and 2 contained the highest numbers of dm-CpGs, while chromosome 14, 15 had the lowest. Such differences are expected given the large sizes of chromosomes 1 and 2. To size-normalize for all chromosomes, we calculated the methylation frequency/Mb for each chromosome to compare the net differential methylation. The size-normalized DNA methylation frequencies indicated that chromosome 20 has the highest differential methylation frequency (14.76 dm-CpGs/Mb) while chromosome 18 has the lowest (0.82 dm-CpG/Mb), as shown in **Table 2** and **Figure 1B**. Except in chromosome 9, hypermethylated CpG sites were more prominent than hypomethylated sites in all the other chromosomes (**Table 2**). We also observed that chromosomes 10 and 18 were extensively hypermethylated to the extent that the hypermethylation frequencies for these two chromosomes were three times higher than the hypomethylation frequencies (**Figure 1B**, **Figure S4**).

**Table 2:** Differential methylation frequency per mega base-pair (Mb) for each autosomal chromosomes.

	Mb	CpG/Mb	Hyper/Mb	Hypo/Mb	Hyper vs Hypo
chr10	133.8	1.26	1.03	0.22	4.6
chr18	80.37	10.66	8.09	2.58	3.14
chr17	83.26	7.04	5.01	2.03	2.47
chr5	181.54	2.17	1.51	0.66	2.31
chr11	135.09	4.29	2.92	1.37	2.14
chr13	114.36	2.26	1.52	0.73	2.07
chr14	107.04	0.82	0.55	0.27	2.03
chr4	190.21	3.26	2.18	1.08	2.02
chr6	170.81	2.38	1.56	0.81	1.92
chr2	242.19	2.9	1.88	1.02	1.83
chr20	64.44	14.76	9.48	5.28	1.8
chr12	133.28	7	4.19	2.81	1.49
chr21	46.71	14.64	8.54	6.1	1.4
chr19	58.62	12.61	7.27	5.34	1.36
chr16	90.34	6.73	3.81	2.92	1.3
chr15	101.99	1.15	0.65	0.5	1.29
chr8	145.14	3.4	1.86	1.54	1.21
chr22	50.82	3.05	1.65	1.4	1.18
chr7	159.35	2.23	1.19	1.04	1.14
chr1	248.96	4.29	2.26	2.03	1.11
chr3	198.3	3.62	1.9	1.72	1.11
chr9	138.39	4.35	1.78	2.56	0.7

To locate genomic regions with high epigenomic perturbations, we calculated dm-CpG frequencies of chromosomal segments in 10 MB sliding windows. Our analysis revealed that chr7:27,000,001–28,000,000 has the highest dm-CpG frequency with the entire region mostly hypermethylated (**Figure 1A**, inner red circle). The region contains several HOX-family genes as HOXA1, HOXA3, HOXA7, HOXA10, HOXA11, and HOXA13.

### Genome-Wide Analysis of Differentially Methylated Regions (DMRs)

The normal differential methylation analysis process does statistical testing for individual CpG sites, but regulatory methylation targets are most commonly clustered into short regions. Clusters of hypermethylated CpG sites in the promoter region of a gene are usually associated with epigenetic silencing of the gene (Jones and Baylin, 2002). Differentially methylated regions (DMRs) comprise multiple consecutive methylated CpG sites with at least two dm-CpGs, therefore detecting DMRs is more biologically relevant (Weaver et al., 2004; Bert et al., 2013).

In all, we identified 779 DMRs across the genome in PDAC. Chromosome 7 showed the highest (74) and chromosome 21 showed the lowest (6) DMRs (**Table S3**). The DMRs were of different lengths, ranging from 3bp to ∼11kb. There were 116 short (<100 bp) DMRs, 84 long (>2 kb) DMRs. The number of dm-CpGs within DMRs ranges from 2 to 45. These DMRs also overlap with the promoters of several HOX-family genes (**Table S3**). Examples of DMRs showing contrasting methylation patterns between normal and tumor samples on chromosome 9 and chromosome 2 are presented in **Figure S5**.

### Differential Gene Expression Analysis

**Figure 2:**
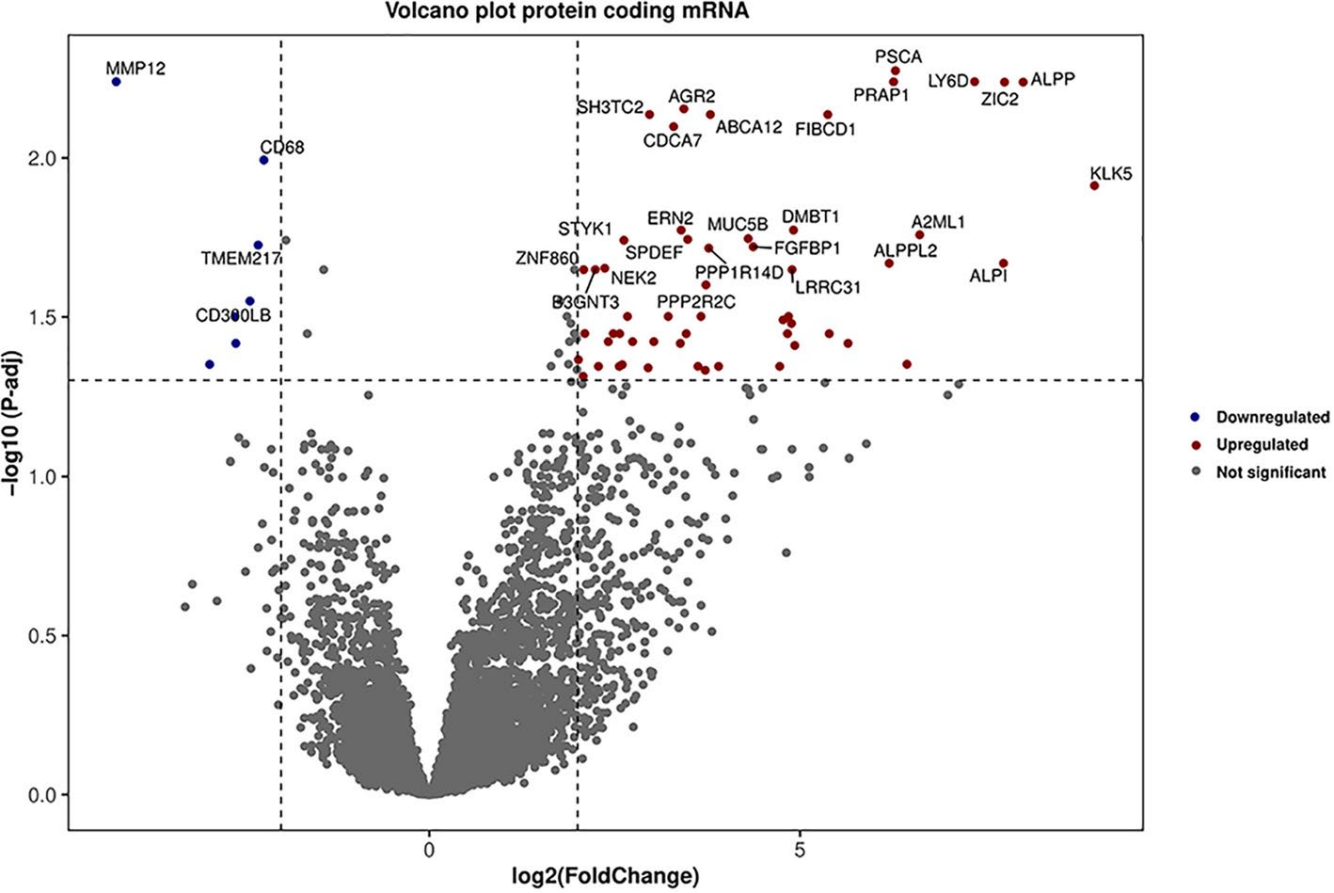
Volcano plot for the differentially expressed genes. Genes which are in red and blue colors are highly upregulated and downregulated, respectively in PDAC. Vertical and horizontal dot line represents a cutoff point for log fold-change *p*-value respectively.

HTSeq read-counts for 146 PDAC patient tumors and three normal samples were downloaded from TCGA and differential gene expression analysis was performed on them using DESeq2 package. Trimmed mean of M-values (TMM) normalization was employed to account for library size variations among samples (Robinson and Oshlack, 2010). We identified 90 differentially expressed genes (80 protein-coding, seven lncRNA, two antisenses, and one Ig-V gene) after adjusting to *p*-value < 0.05 (significance corrected using the Benjamini-Hochberg method) (**Figure 2**, **Table S4**). From the 147 tumors and three normal samples, 10 differentially expressed miRNAs were found (**Table S4**).

### Promoter DNA Methylation and Gene Expression Correlation Analysis

We used Spearman’s test to examine correlations between promoter DNA methylation (within 1.5kb from TSS) and gene expression using the R function, *cor.test*. Correlations that had rho values of ≥ |0.25| and BH adjusted *p*-values of < 0.005 were taken as significant. We observed correlations of 30,619 promoter CpGs with the expression of 8,932 genes, the majority of which were negatively correlated (25,077 CpGs with 7,518 genes), with only a minority (5,605 CpGs with 2,937 genes) showing positive correlations. At higher rho threshold values (|0.5|) and low FDR (<0.005), we observed correlations of 4,971 CpGs with the expression of 1,744 genes, out of which most (4,568 CpGs with 1,602 genes) were negatively correlated and fewer (407 CpGs with 212 genes) were positively correlated (**Table S5**, **Figure S6**).

Similar Spearman’s analyses were performed for finding correlations between CpGs and lncRNAs. We identified 1,216 CpGs that were significantly correlated with 442 lncRNAs, out of these the great majority (1,039 CpGs with 368 lncRNAs) were negatively correlated and fewer (177CpGs with 95 lncRNAs) were positively correlated. At higher thresholds (rho ≥ |0.5| and BH adjusted *p*-value ≤ 0.005), we observed that 199 CpGs were correlated with 84 lncRNAs, out of which 174 CpGs showed negative correlations with 72 lncRNAs, and 25 CpGs were positively correlated with 12 lncRNAs (**Table S5**).

### Pathway Enrichment Analysis

Analyses of differentially methylated CpGs using the Bioconductor missMethyl pathway tool indicated the enrichment of several KEGG pathways (**Table 3**). Several critical cancer-related pathways such as MAPK signaling, Rap1 signaling, calcium signaling were shown in the list. We also observed the enrichment of the nicotine addiction pathway as corroborated by the fact that these patients were cigarette smokers (**Table S3**). In case of differential expression, we observed only 80 differentially expressed genes and no significant pathways were enriched from that list of genes.

**Table 3:** KEGG pathway analysis for differentially methylated genes. We used *missMethyl* tool for pathway analysis. For each enriched pathways, N is the total gene in given pathways, DN is the number of mapped genes in hg38 against differentially methylated CpGs, P.DM is the *p*-value, and FDR is the BH adjusted *P*-value.

Pathway	N	DM	P.DM	FDR
Neuroactive ligand-receptor interaction	252	90	1.37E-09	4.51E-07
Calcium signaling pathway	173	73	5.36E-07	8.84E-05
Rap1 signaling pathway	203	77	3.40E-05	0.00374
Nicotine addiction	36	20	0.00014	0.01117
MAPK signaling pathway	283	96	0.00031	0.02041
cAMP signaling pathway	191	66	0.00037	0.02043
Salivary secretion	81	31	0.00064	0.03027
Circadian entrainment	95	40	0.00102	0.03834
Morphine addiction	88	38	0.00105	0.03834
Mucin type O-glycan biosynthesis	29	14	0.00132	0.04339

### Survival Analysis

We used an in-house R code to perform survival analysis base on the DNA methylation, gene expression, miRNA, and lncRNA results. This R code uses the R tools, *survival*, and *survMiner* in the background and performs the Cox regression and log-odd tests, and generates KM-plots for CpGs, genes, miRNAs, and lncRNAs—all in the context of significant difference in patient survival in the high and low expression groups. In Cox regression analysis, we used low expression and methylation group of samples as reference. The hazard ratio (HR) > 1 indicates high expression group patients have low survival and <1 suggests high survival.

We conducted survival analysis of PDAC patients with respect to differentially methylated CpGs (*p*-values for both log-odd and Cox regression ≤ 0.05). The results identified 439 CpGs that may have survival roles. Out of these, 80 showed survival relationship at a stringent selection criterion (*p*-value ≤ 0.01). In contrast, survival analysis of the gene expression data indicated 1,954 genes that may influence PDAC patient survival with *p*-value ≤ 0.05 (**Table S5**). When we reduced survival *p*-value cutoff to 0.01, this gene number goes down to 518. Similarly, we observed 236 lncRNAs which correlated with survival at *p*-value ≤ 0.05, whereas this number came down to 74 at *p*-value cutoff of 0.01. For miRNA, these numbers were 25 at *p*-value ≤ 0.05 that were reduced to 7 at *p*-value ≤ 0.01.

### Correlative Analysis of Gene Expression and Survival

Genes and genomic regulatory loci that are differentially expressed and correlated with patients’ survival could be important for understanding the initiation and progression of PDAC. Integrative analysis of patient survival and differential expression identified 17 genes that passed our tests at BH adjusted *P*-value ≤ 0.05 for both differential expression and patient survival or five genes when the thresholds were decreased to 0.01 for both DEG and survival analysis (**Table 4**). In these tests, we did not observe any differentially expressed lncRNAs that correlated with PDAC patient survival.

**Table 4:** List of probable prognostic gene/miRNA biomarkers for pancreatic ductal adenocarcinoma. List of genes and miRNA which have very low *p*-value in survival and *DESeq2* differential gene expression analysis, and high area under curve (AUC).

Gene	log2FC (DESeq)	*P*-value (DESeq)	*P*-adj (DESeq)	*P*-value (log Rank)	*P*-value (Cox)	Beta (Cox)	HR (95% CI)	AUC
**ASPM**	1.986477	0.000131	0.036978	0.05	0.052	0.43	1.5 (1–2.4)	0.96
**B3GNT3**	2.237597	4.19E-05	0.022447	0.011	0.012	0.57	1.8 (1.1–2.8)	0.93
**BMF**	-1.41789	4.63E-05	0.022447	0.03	0.032	-0.47	0.62 (0.4–0.96)	0.89
**CD300LB**	-2.4129	6.62E-05	0.028185	0.008	0.0091	-0.58	0.56 (0.37–0.87)	0.83
**CD68**	-2.22506	8.73E-06	0.010149	0.035	0.037	-0.46	0.63 (0.41–0.97)	0.92
**CENPF**	1.890233	0.000144	0.037802	0.018	0.019	0.52	1.7 (1.1–2.6)	0.96
**DEPDC1B**	2.074024	0.000251	0.048577	0.005	0.0054	0.63	1.9 (1.2–2.9)	0.95
**DMBT1**	4.911577	1.72E-05	0.016867	0.023	0.024	-0.51	0.6 (0.39–0.94)	0.89
**DTL**	1.640861	0.000222	0.045194	0.026	0.028	0.49	1.6 (1.1–2.5)	0.97
**ERCC6L**	1.957758	4.48E-05	0.022447	<0.001	0.00056	0.79	2.2 (1.4–3.5)	0.97
**FAM111B**	1.768394	6.58E-05	0.028185	0.022	0.024	0.51	1.7 (1.1–2.6)	0.96
**HIST1H2BC**	2.740626	0.000145	0.037802	0.003	0.0039	0.66	1.9 (1.2–3)	0.95
**HIST1H2BJ**	2.949891	0.000228	0.045665	0.032	0.034	0.48	1.6 (1–2.5)	0.93
**HIST1H3H**	3.383461	0.000154	0.038262	0.016	0.017	0.55	1.7 (1.1–2.7)	0.91
**KIF4A**	1.855617	8.42E-05	0.031507	0.013	0.014	0.55	1.7 (1.1–2.7)	0.95
**NEK2**	2.364569	3.95E-05	0.022212	0.001	0.0019	0.71	2.0 (1.3–3.2)	0.95
**RASSF4**	-1.6378	0.000121	0.035665	0.046	0.048	-0.43	0.65 (0.45–1.0)	0.97
***hsa-mir-196b***	3.542765	0.000697	0.020469	0.002	0.0022	0.69	2.0 (1.3–3.1)	0.83

Log2FC, log2 fold change; HR, hazard ratio; 95% CI, upper and lower 95% confidence interval values of hazard ratio (HR), and beta is β coefficient of a given variable for the Cox regression analysis.

**Figure 3:**
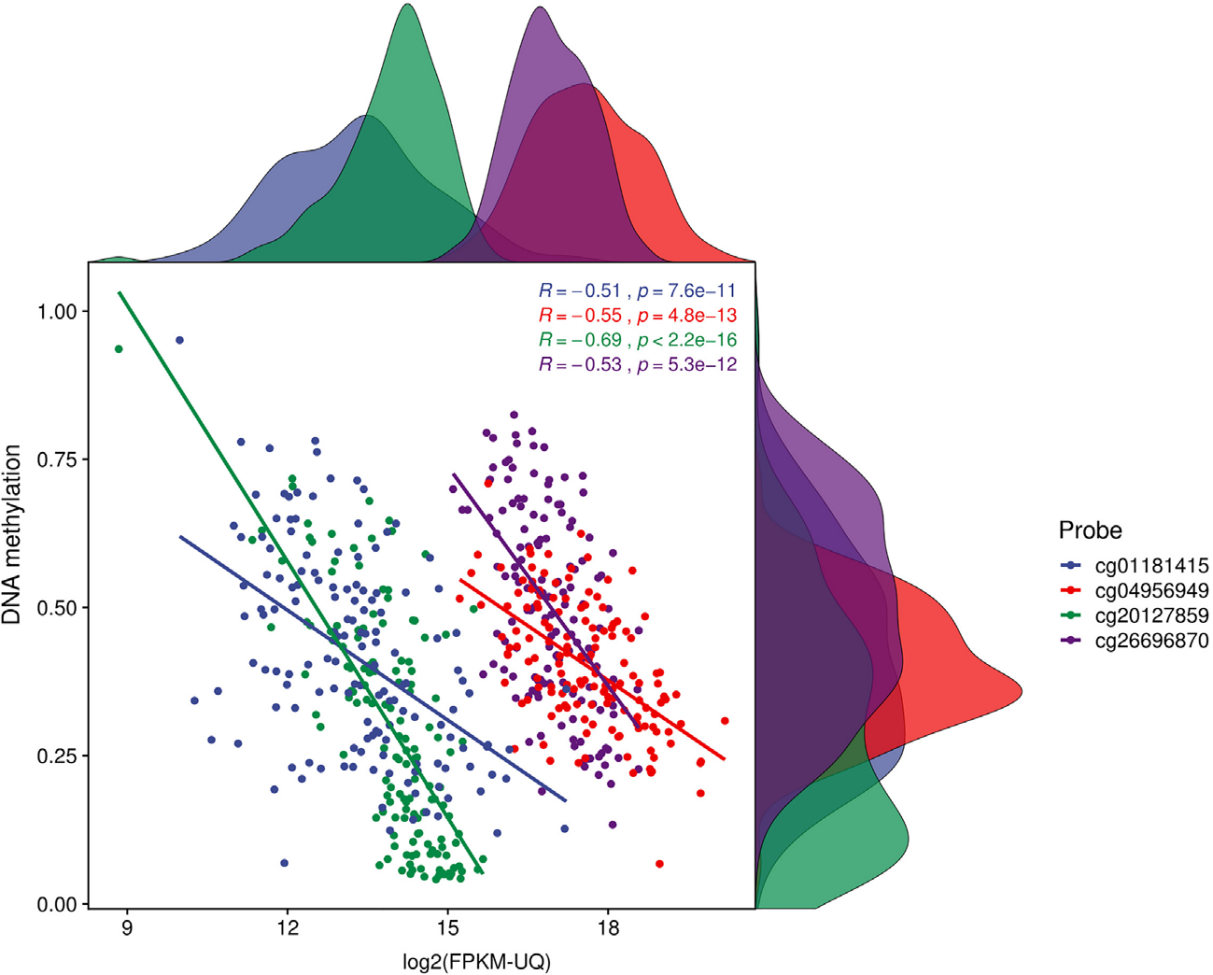
Correlation plot for survival associated CpGs. We used CpGs which have survival p < = 0.01 and Spearman correlation > 0.5 (*p*-value < 0.005). This plot is for four promoter CpGs which are negatively correlated with genes expression and also strongly associated with patients’ survival. Distribution of DNA methylation and gene expression in PDAC patients on the right side and top respectively.

Further analysis of genes that have dm-CpGs in the promoter regions (∆β ≥|0.2|, FDR < 0.005) and showing a negative correlation in corresponding gene expression (rho ≤ -0.5, FDR < 0.005) showed that a total of 93 CpGs have a significant difference (*p*-value ≤ 0.05) in survival between high and low patient groups. This number further goes down to 4 if we use *p*-value ≤ 0.01 in the survival analysis (**Figure 3**).

In the case of lncRNA, we observed that three promoter dm-CpGs showing a negative association with lncRNA expression have a role in overall patient survival (*p*-value ≤ 0.05). This number goes down to two if we further reduce survival *p*-value to 0.01. List of these CpGs with survival details are shown in **Table S7**.

### Analysis of Genes of Mucin Family

**Figure 4:**
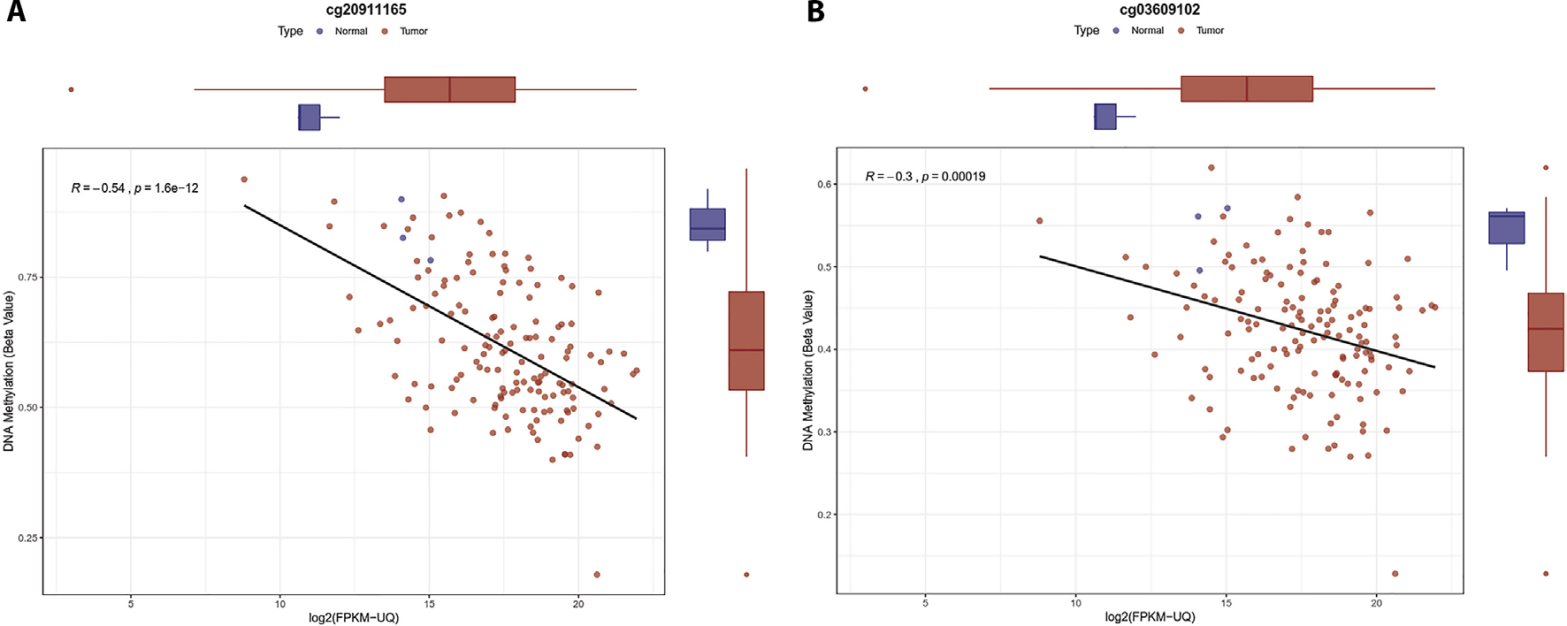
Correlation plot for the MUC5B promoter methylation sites. Boxplot for gene expression and DNA methylation on top and right side respectively, tumor samples are in red and normal samples in blue color. **(A)** Correlation plot and boxplot for cg20911165. **(B)** Correlation plot and boxplot for cg03609102.

Our DEG analysis showed that MUC2, MUC5B, and MUC13 were significantly upregulated in PDAC (**Table S8**). MUC1, MUC6, and MUC16 showed overexpression but it was not statistically significant (BH adjusted *P*-value > 0.05). We noted that MUC5B, which was overexpressed in PDAC (BH adjusted *P*-value = 0.018) has also two hypomethylated CpGs (cg20911165 and cg03609102) in its promoter region, which also showed a negative correlation with MUC5B expression (**Figure 4**). We also observed that expression of MUC1, MUC3, MUC4, MUC6, MUC15, MUC17, MUC20, and MUC21 genes was negatively correlated with the promoter methylation (**Table S5**).

## Discussion

Alterations in the promoter DNA methylation, as well as miRNA and lncRNA expression, play critical roles in cancer biology by up- or downregulating gene expression (Merlo et al., 1995; Ramachandran et al., 2016). DNA methylation pattern alterations can serve as useful biomarkers for distinguishing tumors from normal samples (Oh et al., 2013). Two previous studies by (Sato et al., 2008) and (Tan et al., 2009) had explored DNA methylation patterns in pancreatic cancer. Sato et al. used methylation-site specific PCR, and Tan et al. used GoldenGate methylation cancer panel array. Both of these technique have limited genome coverage and sensitivity. In addition, those studies used formalin-fixed paraffin embedded samples, xenografts, and pancreatic cancer cell lines, which might affect the quality of the results. On the other hand, the current study is based on TCGA Illumina HumanMethylation450 chip from fresh tissue samples, which has higher genome coverage with greater consistency and accuracy. Our study is more comprehensive, since we scoped for differential methylation, differential gene expression, differential miRNA, differential lncRNA in a genome-wide manner, and we also correlated these results with patient survival. To avoid gender bias, we excluded all CpG probe and gene expression data from X and Y chromosomes. Our results demonstrated that all chromosomes had dm-CpGs in PDAC (**Figure 1A**, **Table 2**). CpG islands, promoter, and their proximal regions had more hypermethylated CpG sites compared to regions away from islands and promoters (**Figure 1C**, **Table 1**, **Figure S6**). We observed that several chromosomal regions which have a high frequency of dm-CpGs are also a region which is differentially methylated.

In this study, CpG sites in the zinc finger protein 154 (ZNF154) promoter region were hypermethylated and showed a negative correlation with ZNF154 gene expression. We found that promoter of ZNF158 overlap with a region which has the highest differential methylation frequency in chromosome 19. The survival analyses indicated that the cg03234186 high methylation group patients had a low overall survival (HR = 1.7) in PDAC (**Table 5**). ZNF154 hypermethylation is a urine-based prognostic biomarker for bladder cancer, where hypermethylation correlates with recurrence-free survival of the patients (Reinert et al., 2012). ZNF154 hypermethylation may also be a blood-based prognostic biomarker for solid tumors (Sanchez-Vega et al., 2013; Margolin et al., 2016). Recently, Zhang *et al*. located CpG hypermethylations at ZNF154 promoter (cg03234186, cg12506930, cg26465391) by studying the TCGA prostate cancer archive. Hypermethylation downregulates ZNF154 expression and survival analysis suggest that hypermethylation of this site is associated with poor survival of patients (Zhang, Shu et al., 2018).

**Table 5:** List of probable prognostic DNA methylation biomarkers for pancreatic ductal adenocarcinoma.

CpG	log-rank	HR (95% CI)	*P*-value Cox	Correlation	*P*-value	*P*-adj	AUC
cg02587316	0.029	1.8 (1.1–3.2)	0.032	-0.56	<1.OE-21	<1.0E-21	0.95
cg18630667	0.012	2 (1.1–3.4)	0.014	-0.56	<1.0E-21	<1.0E-21	0.96
cg05020604	0.015	1.9 (1.1 3.3)	0.017	-0.55	<1.0E-21	<1.0E-21	0.96
cg03234186	0.043	1.7 (1.0–2.9)	0.046	-0.67	<1.0E-21	<1.0E-21	0.90

AUC, area under curve; HR, hazard ration; P-value Cox, the P-value for cox regression analysis. P-value and P-adj are the raw P-value and BH adjusted P-value respectively for the Spearman rank correlation.

**Figure 5:**
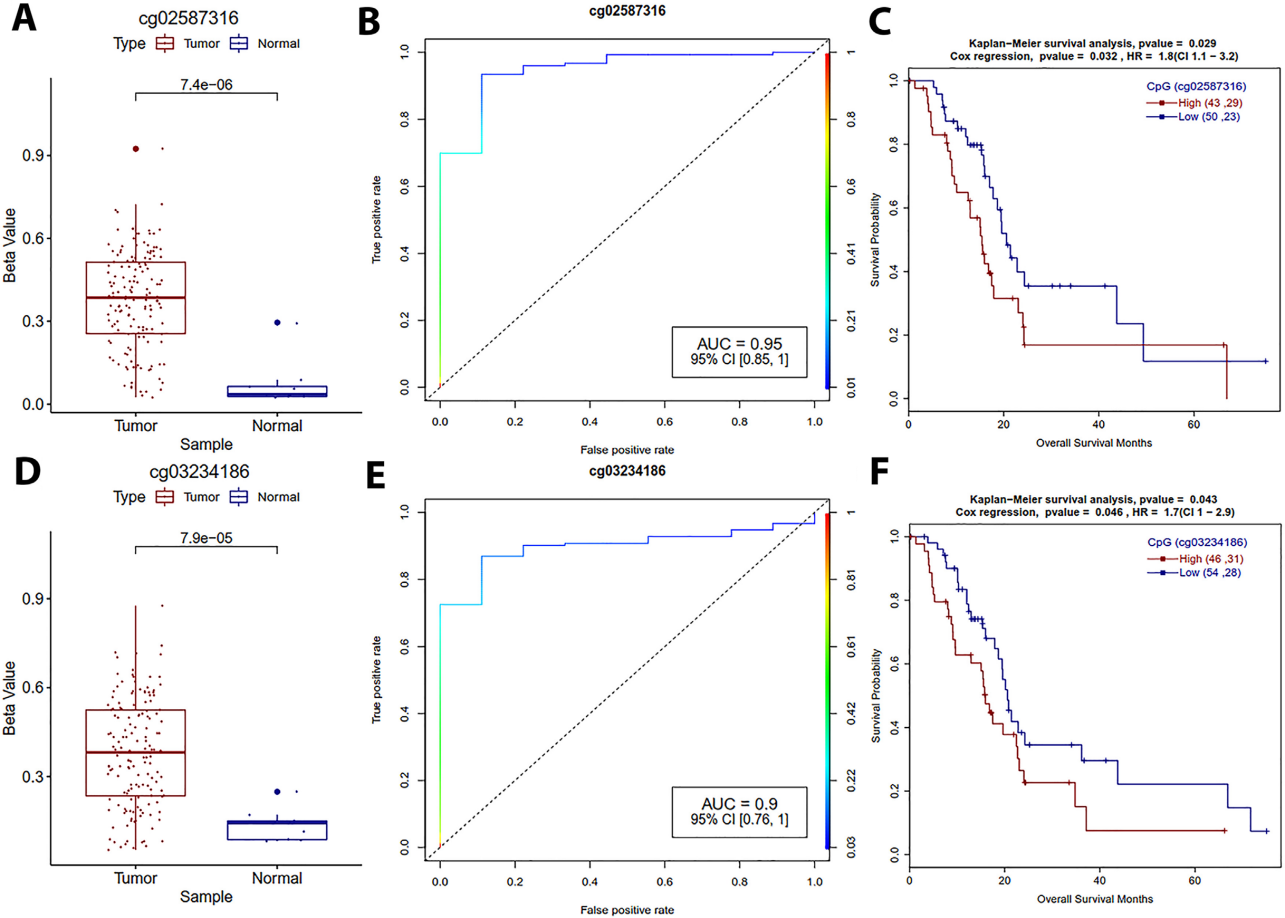
Survival plots for zinc finger gene promoter DNA methylation sites which are associated with PDAC patients’ survival. **(A, D)** Boxplot for cg02587316 and cg03234186 DNA methylation distribution for tumor and normal samples with Welch *t*-test. **(B, E)** ROC plot for cg02587316 and cg03234186 for the generalized linear model classifier. **(C, F)** Survival plot for high vs low methylation group for cg02587316 and cg03234186 with a *p*-value for Kaplan–Meier plot (log-rank test) and Cox proportional hazards model.

KRAB zinc-finger tumor suppressor ZNF382 expression is suppressed by promoter methylation in esophageal squamous cell carcinoma (Zhang, Xiang et al., 2018). In PDAC, we identified hypermethylations in five CpG sites in the ZNF382 promoter region, which are negatively correlated with gene expression. Logistic regression-based classification showed an AUC of 1.0 for all these CpGs. Hypermethylation of (cg02587316, cg18630667, and cg05020604) was associated with low survival of PDAC patients (**Table 5**). Above findings suggest that methylation of cg03234186 (ZNF154), and cg02587316, cg18630667, cg05020604 (ZNF382) have the potential to serve as prognostic biomarkers for PDAC (**Figure 5**).

The differentially expressed miRNAs include hsa-mir-196-a1/2 and hsa-mir-196b, both of which are HOX-cluster embedded miRNA members of the evolutionarily conserved miR-196 gene family (Mansfield and McGlinn, 2012; Fantini et al., 2018). The hsa-mir-196-a1 gene is located in the intergenic region between HOXB9 and HOXB13 on human chromosome 17; the hsa-mir-196a-2 between HOXC9 and HOXC10 on chromosome 12, and the hsa-mir-196b is on chromosome 7. HOX genes such as HOX-B7 (Braig et al., 2010), HOXB8 (Yekta et al., 2004), and HOXA9 (Li et al., 2012) are targets of the miR-196 family. MiR-196b directly targets HOXA9, whose overexpression is associated with bad prognosis in leukemia (Li et al., 2012). The hsa-mir-196a-regulated HOX-B7 expression has a role in melanoma (Braig et al., 2010), it would be worth investigating the role of HOX-cluster gene regulation by miRNA and/or promoter methylations in pancreatic cancers.

**Figure 6:**
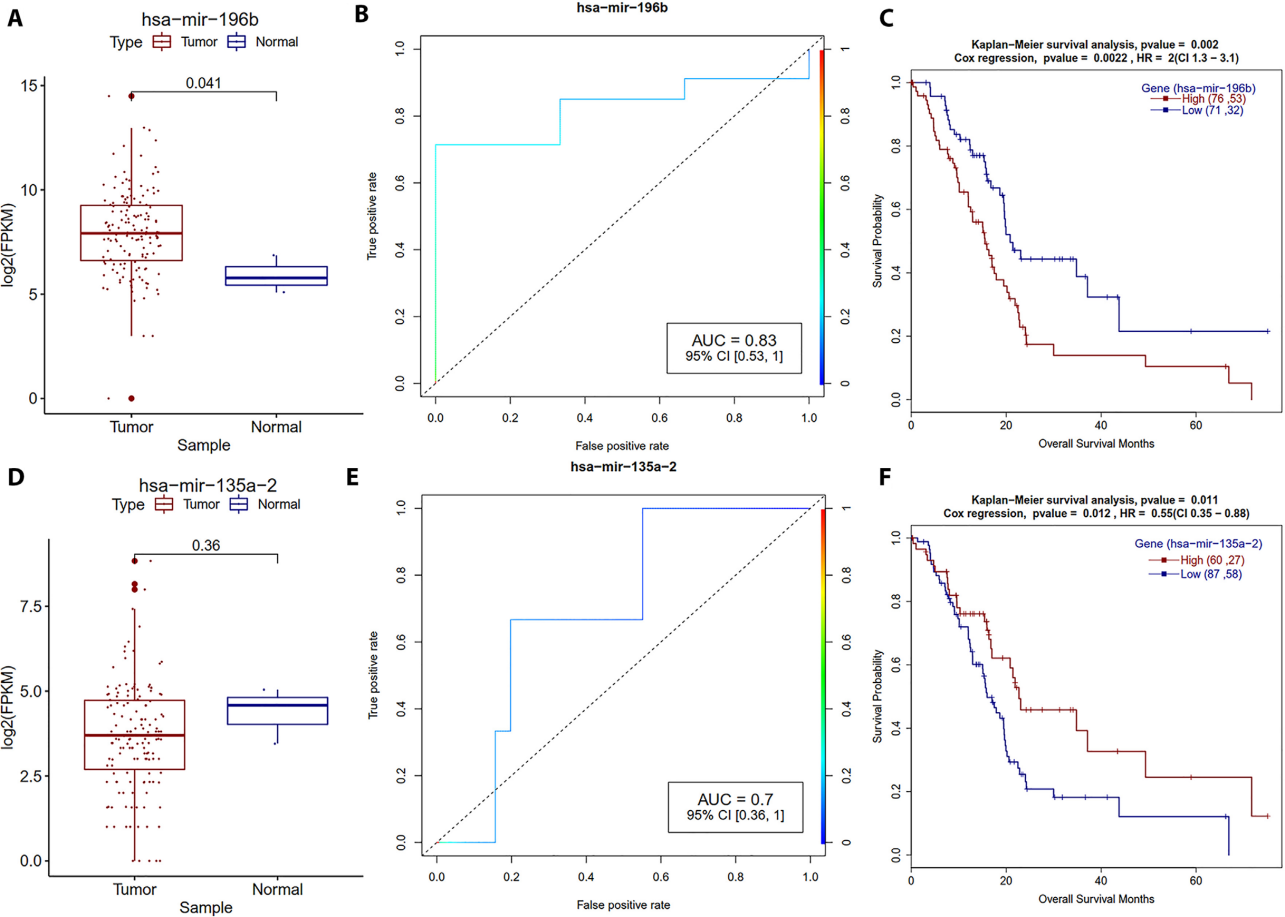
Survival plots for the miRNA which are strongly associated with the PDAC patients’ survival. **(A, D)** Boxplot for miR-196b and miR-111 miRNA expression distribution for tumor and normal samples with Welch *t*-test. **(B, E)** ROC plot for miR-196b and miR-111 miRNA for the generalized linear model classifier. **(C, F)** Survival plot for high vs low methylation group for miR-196b and miR-111 miRNA with a *p*-value for Kaplan–Meier plot (log-rank test) and Cox proportional hazards model.

Hsa-mir196-b has been reported as a biomarker for digestive tract cancers (Lu et al., 2016) and familial pancreatic cancer (Slater et al., 2014). Multiple studies indicate that hsa-mir196-b overexpression is bad for the cancer patient. For example, hsa-mir196-b overexpression is associated with poor prognosis in gastric cancer (Lim et al., 2013; Ge et al., 2014), and is also associated with accelerated invasiveness in epithelial ovarian cancer (Chong et al., 2017). Kanno et al., (2017) reported that hsa-mir-196b overexpression might be a prognostic biomarker for a bad outcome. In our current study, we also found that PDAC patients with hsa-mir-196b overexpression showed worse survival (**Table 4**, **Figure 6**), which further corroborates the role of hsa-mir-196b as a biomarker for PDAC.

MiR-125a is a tumor suppressor that induces apoptosis, mitochondrial energy disorder, and cellular migration through suppressing mitochondrial fission, and play an important role in pancreatic cancer (Pan et al., 2018). Metastatic colorectal cancer patients treated with bevacizumab in combination with FOLFOX have better progression-free survival (Kiss et al., 2017). In the current study, we observed that hsa-mir-125a is overexpressed but *P*-value was not significant, however, univariate Cox regression analysis suggested that patients with higher expression of mir-125a had a better overall survival (HR = 0.57) (**Table S6**). This finding suggests that hsa-mir-125a might be useful as a prognostic biomarker for PDAC.

Hsa-mir-135a-2 is a precursor of hsa-mir-135a; univariate log-rank test (*P*-value = 0.01) and Cox-regression analysis (HR = 0.55) suggest that higher expression is associated with better overall survival of PDAC patients. Cheng *et al*. reported that mir-135a is a metastasis inhibitor, and they observed similar survival trends in gastric cancer cell line data (Cheng et al., 2017). In our study, we also observed that hsa-mir-3200 expression is associated with good prognosis of PDAC (HR = 0.5) (**Table S6**).

From the survival analyses of protein-coding genes in PDAC, we observed 518 genes that had significant correlations with patient survival both in high and low expression cohorts. The aryl hydrocarbon receptor nuclear translocator like 2 (ARNTL2) gene, which codes for a helix-loop-helix transcription factor, was the most significant among all. Overexpression of this gene was reported to predict poor outcome for lung adenocarcinoma patients (Brady et al., 2016). To our knowledge, the role of ARNTL2 in PDAC was not explored before, and the current study showed that ARNTL2 overexpression had a strong association with poor survival (HR = 2.2) in PDAC patients.

In the contrary, overexpression of certain genes was also found to help extend patient survival. Overexpression of CELF2 and EFR3B were correlated with better PDAC patient survival (**Table S6**). CELF2 is a tumor suppressor (Subramaniam et al., 2011; Ramalingam et al., 2012), and EFR3B contributes to the control of the phosphorylation state and could affect the responsiveness of G-protein-coupled receptors in higher eukaryotes (Bojjireddy et al., 2015). The role of EFR3B is mammalian is still unexplored, nevertheless, our results indicated that its expression is a key indicator of patient survival.

The abnormal expression of many long non-coding RNAs (lncRNAs) has been reported as effectors in the progression of various cancers. Some of these lncRNAs may be useful as diagnostic indicators and anti-cancer targets (Petrovics et al., 2004; Gutschner et al., 2013). We explored whether lncRNAs were involved in PDAC and whether we can find any indication for their utility for the diagnosis and treatment of PDAC. However, none of their expression patterns were correlated with patient survival. It is possible that we needed more than the three tumor-adjacent normal samples for examining lncRNAs. Unfortunately, the present TCGA database has expression values for only three lncRNAs. However, we did find a few lncRNA expression and survival correlations at low *P*-value thresholds (*P*-value ≤ 0.05) that could be further tested for their role in patient survival (**Table S6**).

**Figure 7:**
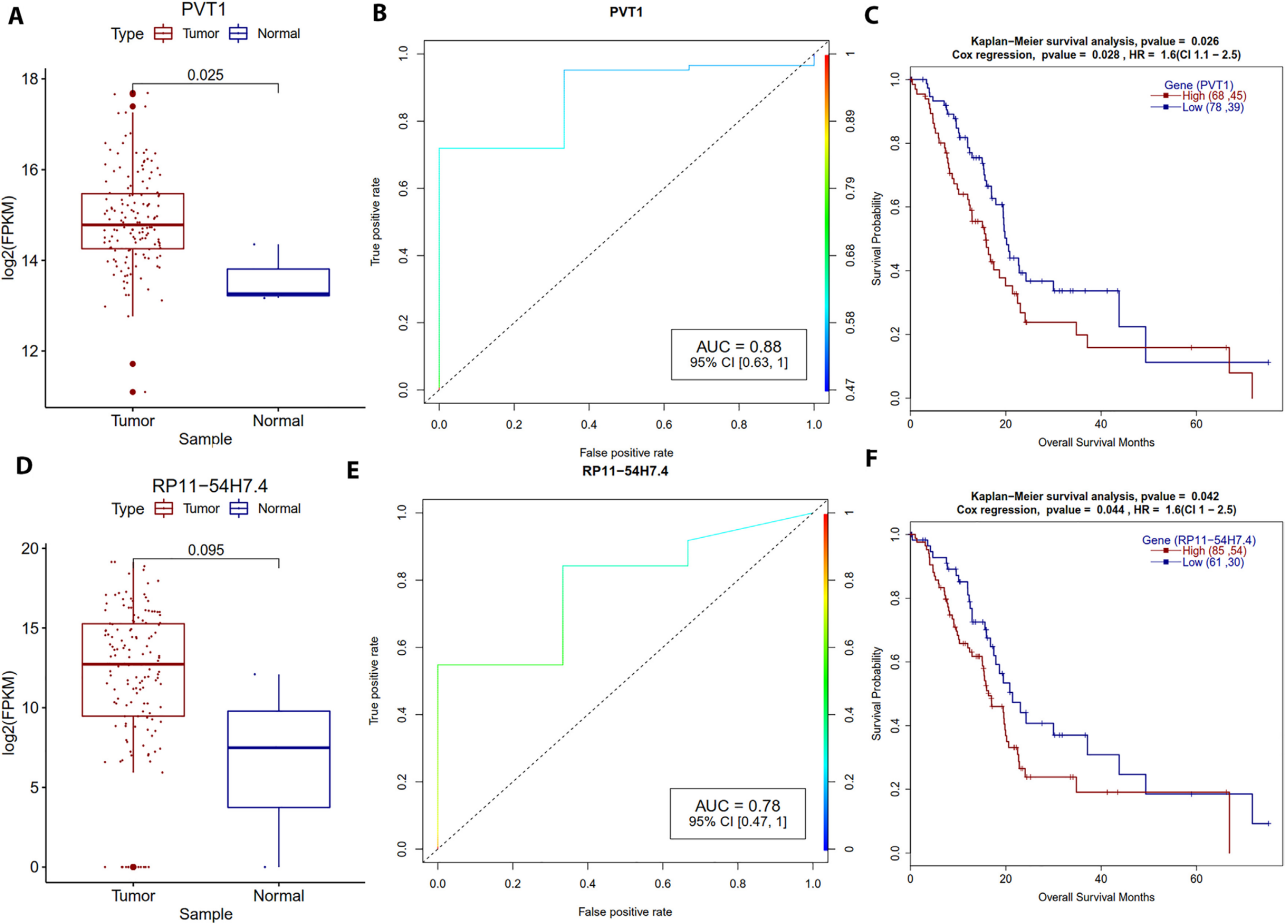
Survival plots for the lncRNA which are strongly associated with the PDAC patients’ survival. **(A, D)** Boxplot for PVT1 and RP11-54H7.4 lncRNA expression distribution for tumor and normal samples with Welch *t*-test. **(B, E)** ROC plot for PVT1 and RP11-54H7.4 lncRNA for the generalized linear model classifier. **(C, F)** Survival plot for high vs low methylation group for PVT1 and RP11-54H7.4 lncRNA with a *p*-value for Kaplan–Meier plot (log-rank test) and Cox proportional hazards model.

LINC00941 is an epigenetically-silenced lncRNA found in pan-cancer TCGA data analysis (Wang et al., 2018). In our study, we found that LINC00941 is overexpressed (*P*-value = 0.02) and that high expression correlated with poor prognosis (HR = 1.8). PVT1 is another lncRNA, which is upregulated in lung cancer and plays a crucial role in lung cancer progression (Li et al., 2018). In our study, PVT1 also turned up overexpressed (*P*-value = 0.009) and correlated with poor PDAC patient survival (HR = 1.60), logistic regression classification AUC is 0.88 (**Figure 7**). Therefore, PVT1 may prove useful as a potential biomarker for PDAC therapy. RP11-54H7.4 is another overexpressed lncRNA in the TCGA database that was reported as a candidate biomarker for lung squamous cell carcinoma prognosis (Tang et al., 2017). We also observed elevated expression of RP11-54H7.4 (not significant), and high expression group PDAC patients had worse survival (HR = 1.6) (**Figure 7**).

A few other lncRNAs had contributory roles in PDAC patient survival, but they did not differentially express. The cancer susceptibility candidate 11 (CASC11) lncRNA is among them. Based on a knockdown study, CASC11 is thought to have a promoting role in colorectal cancer growth and metastasis (Zhang et al., 2016). Our current study showed that CASA11 overexpression associated with low survival. The antisense lncRNA of GATA6 (GATA6-AS) interacts with an epigenetic regulator LOXL2 to regulate endothelial gene expression *via* changes in histone methylation (Neumann et al., 2018). Our study showed that GATA6-AS overexpression correlated with poor prognosis of PDAC patients. A second similar lncRNA (GATA6-AS1) also was overexpressed and correlated with poor survival of PDAC patients (HR = 0.5) (**Table S6**).

Regarding protein-coding genes (**Table 4**), our study found 17 differentially expressed genes but five of them were identified at a stringent *P*-value of ≤ 0.01 that also correlated with PDAC patient survival. Expression of ASPM, Nek2, B3GNT3, DMBT1, and DEPDC1 is associated with better survival of PDAC patients in this study. ASPM (abnormal spindle-like microcephaly associated) is an oncogene that promotes tumor aggression in PDAC, and overexpression is associated with poor prognosis (Wang et al., 2013). We also observed that the ASPM overexpressing patient group showed low survival. NIMA-related kinase 2 (Nek2) is a serine/threonine kinase that plays a critical role in mitosis. Nek2 was reported as a prognostic biomarker for lung cancer (Shi et al., 2017), and knockdown of Nek2 gene with siRNA in xenograft mice decreased tumor size and increased survival for liver metastasized pancreatic cancer (Kokuryo et al., 2016). This gene was also reported as a prognostic biomarker for PDAC, as patients with high Nek2 expression showed shorter survival (Ning et al., 2014). In the current study, we observed a similar trend, our logistic regression model analysis also suggests that Nek2 expression may be a distinctive trait in PDAC vs. normal samples (AUC = 0.95). Our finding further reconfirms that Nek2 is a potential prognostic biomarker of PDAC.

**Figure 8:**
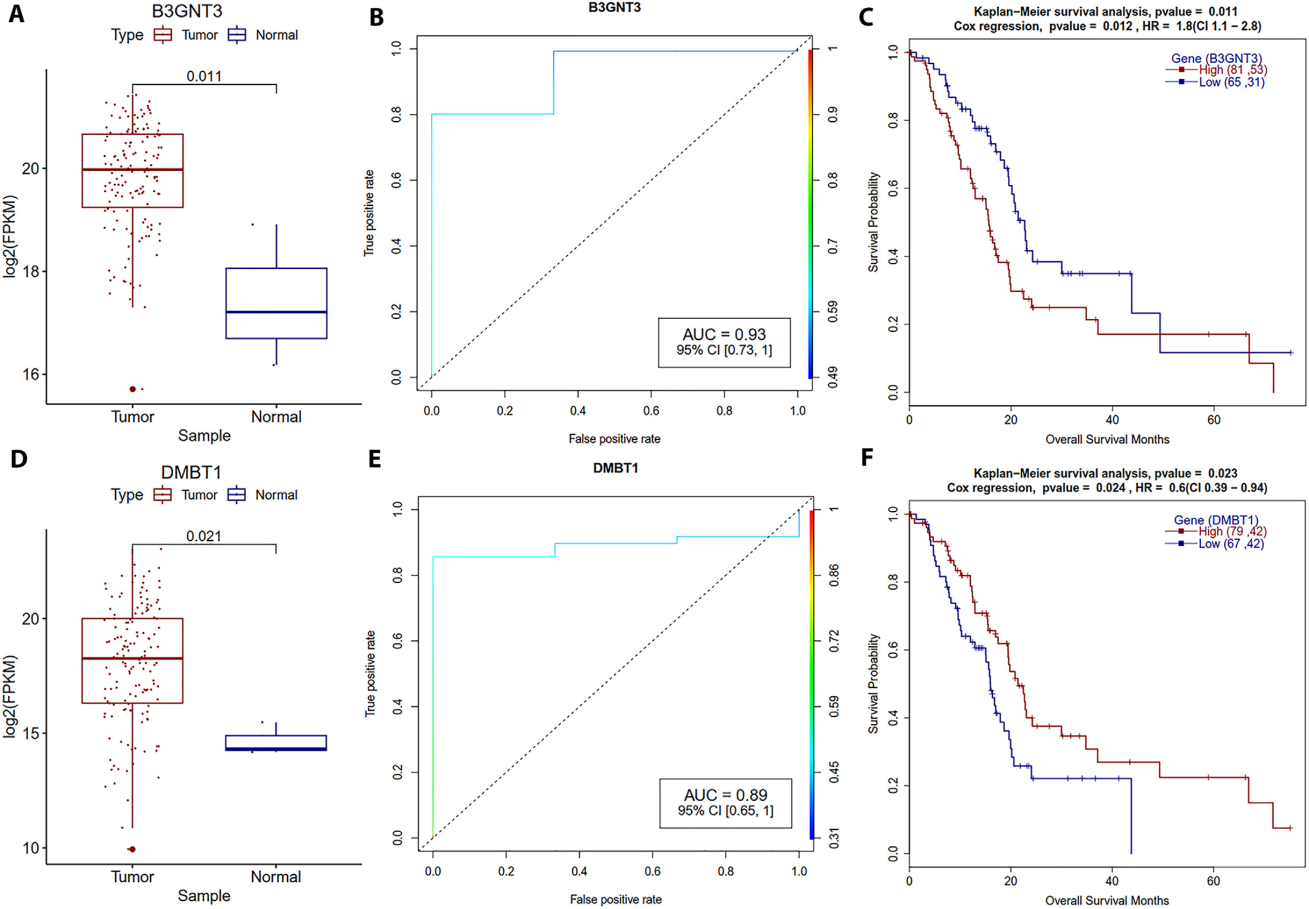
Survival plots for the genes which are strongly associated with the PDAC patients’ survival. **(A, D)** Boxplot for B3GNT3 and DMBT1 gene expression distribution for tumor and normal samples with Welch *t*-test. **(B, E)** ROC plot for B3GNT3 and DMBT1 for the generalized linear model classifier. **(C, F)** Survival plot for high vs low expression group for B3GNT3 and DMBT1 with a *p*-value for Kaplan–Meier plot (log-rank test) and Cox proportional hazards model.

We observed that overexpression of B3GNT3 (beta-1,3-N-acetylglucosaminyltransferase-3) is associated with shorter survival in PDAC (**Figure 8**). High AUC for the logistic regression model (AUC = 0.93) and low *P*-value with the high hazard ratio in Cox regression analysis suggests that this can be a potential prognostic biomarker for PDAC. Previous reports also confirmed that B3GNT3 overexpression was associated with shorter survival of patients in the cervical (Zhang et al., 2015) and non-small lung cell (Gao et al., 2018) cancers. Similarly, overexpression of the DEP domain containing 1 (DEPDC1) is associated with shorter overall survival of PDAC patients. Overexpression of DEPD1B is already reported in several types of human cancers (Su et al., 2014; Huang et al., 2017), we also observed overexpression in PDAC. High classification AUC (0.95) and Cox regression HR (1.9) suggest that it’s a good candidate for prognostic biomarker in PDAC (**Table 4**). These findings suggest that our proposed methodology is working well for detecting known biomarkers, so it can as well detect novel prognostic biomarkers.

On the other hand, overexpression of DMBT1 and Bcl2-modifying factor (Bmf) is shown to improve survival in our study. DMBT1 (deleted in malignant brain tumors 1) expression cohorts have better survival (HR = 0.6) and high logistic regression classification AUC (0.95) suggests its role as a potential biomarker (**Figure 8**). DMBT1 is a tumor suppressor and involved in immune defense and epithelial differentiation in cancer (Mollenhauer et al., 2000). Expression of DMBT1 goes down in breast cancer (Braidotti et al., 2004; Blackburn et al., 2007), we observed a similar trend in our analysis. Pro-apoptotic protein Bmf which regulate the death of CD8 T cells (Hubner et al., 2010), is a probable prognostic biomarker for PDAC (HR = 0.62), samples with high expression Bmf have a good prognosis.

Mucins are high molecular weight glycoproteins with oligosaccharides attached to serine or threonine residues of the mucin core protein backbone that play important roles as diagnostic and prognostic markers for carcinogenesis and tumor invasion (Hollingsworth and Swanson, 2004). We separately analyzed the promoter DNA methylation and mucin gene expression in pancreatic ductal cancer. We observed significant upregulation of MUC2, MUC5B, and MUC13 in PDAC. MUC5B and MUC13 overexpressed in pancreatic ductal cancer (Kaur et al., 2013), the MUC5B expression is highly sensitive to change in promoter methylation (Yamada et al., 2011). We observed the hypomethylation of MUC5B promoter CpG cg20911165 and cg03609102 which is negatively correlated with the gene expression (**Figure 4**). We also observed overexpression of MUC2 gene, in general, its expression goes down in PDAC but some report also suggests overexpression of MUC2 (Niv, 2017). Survival analysis of PDAC data reveals that patients which have higher expression of MUC21 have low survival rate (Cox-*P*-value = 0.04, HR = 1.6).

Pathways analysis didn’t observe any significantly enriched pathways for the differentially expressed genes in pathway enrichment analysis, as number of genes is not enough for analysis. But, pathway analysis of loci with dm-CpGs suggested that MAPK signaling, Rap1 signaling, cAMP signaling, cancer signaling, and mucin type O-glycan biosynthesis pathways were enriched. We conjecture that the nicotine and morphine addiction pathway showed up in our analysis because these PDAC patients are current or past smokers (**Table 3**). Many other cancer-related genes showed up differentially expressed in PDAC, including MUC2, MUC5B, MUC13, ALDH3A1, CDCA7, and CCL2. Several histone core proteins were overexpressed in PDAC. Our current study also indicated that HIST1H2BC, HIST1H2BJ, and HIST1H3H were associated with poor survival of PDAC patients (**Table 4**).

## Conclusions

To our knowledge, this study represents the first TCGA-based PDAC methylome data analysis. The DNA methylome of pancreatic ductal cancer showed significant changes from normal samples. Most of hypermethylation taking place within the promoter regions and methylation in the promoter region have a strong association with corresponding gene expression. A 10 Mb region of chromosome 7 has the highest hypermethylation density, and this region harbors a number of HOX cluster genes. MUC family genes and histone core proteins are overexpressed, expression of MUC21 and several histone core HIST1H2AC, HIST1H2BC, and HIST3H2A are also associated with patients’ survival. Role of hsa-mir-196b and Nek2 in PDAC patients’ survival is further reconfirmed. Our analysis reveals that protein-coding genes, ARTNTL2, CELF2, EFR3B, B3GNT3, and long non-coding genes, CASC11, GATA6-AS are potential prognostic biomarkers of PDAC. Promoter methylation of ZNF154 and ZNF382, which were previously reported as early stage urine/blood-based biomarkers have the potential to be prognostic biomarkers for PDAC.

## Data Analysis

All analyses were performed using the R version 3.5.1 (R Development Core Team 2015). We performed differential methylation/expression and survival analysis by using R/­Bioconductor tools. List of tools used for this analysis are available in **Table S1**.

## Author Contributions

NM, SS, and CG are responsible for the study design. NM and SS performed the statistical analysis and generated figures. NM and SS drafted the manuscript and CG edited and improved the manuscript and approved it.

## Funding

The authors thank the Bioinformatics and Systems Biology Core, which receive partial support from National Institutes of Health grants [P20GM103427, P30CA036727].

## Conflict of Interest Statement

The authors declare that the research was conducted in the absence of any commercial or financial relationships that could be construed as a potential conflict of interest.
