# Supplementary material for: Survival Analysis of Multi-Omics Data Identifies Potential Prognostic Markers of Pancreatic Ductal Adenocarcinoma
Source: Front Genet. 2019 Jul 18;10:624. doi: 10.3389/fgene.2019.00624 (PMC6659773; doi:10.3389/fgene.2019.00624)
Supplement: Supplementary file 1 [file DataSheet_1.pdf]

Supplementary Material

Survival analysis of multi-omics data identifies potential prognostic markers of pancreatic ductal adenocarcinoma

Nitish Kumar Mishra, Siddesh Southekal, Chittibabu Guda*

*** Correspondence:** Corresponding Author: babu.guda@unmc.edu

1 Supplementary Data

Supplementary tables are available on FigShare.

Table S2: https://figshare.com/s/2f2359bf82e3a8db118f

Table S3: https://figshare.com/s/90c7a4decd480006ab9f

Table S4: https://figshare.com/s/6033221610cca1fe4fb8

Table S5: https://figshare.com/s/e183e0a487f87bebc3a8

Table S6: https://figshare.com/s/205a5f6ecd86a81314d7

Table S7: https://figshare.com/s/083f83bb641bd4901ec1

2 Supplementary Tables and Figures

2.1 Supplementary Tables

Table S1: List of R/Bioconductor tools and database used in pancreatic ductal cancer data analysis.

Resource	Source	Identifier
**Software and algorithms**		
R	R core team	https://www.r-project.org/
Bioconductor	Bioconductor	https://www.bioconductor.org/
TCGABiolinks	Bioconductor	https://github.com/BioinformaticsFMRP/TCGAbiolinks
BMIQ	R program	https://sourceforge.net/projects/bmiq/
Limma	Bioconductor	https://bioconductor.org/packages/release/bioc/html/limma.html
ROCR	R package	https://rocr.bioinf.mpi-sb.mpg.de/
Gtrellis	Bioconductor	https://github.com/jokergoo/gtrellis
DMRcate	Bioconductor	https://www.bioconductor.org/packages/release/bioc/html/DMRcate.html
DESeq2	Bioconductor	https://www.bioconductor.org/packages/release/bioc/html/DESeq2.html
clusterProfiler	Bioconductor	https://github.com/GuangchuangYu/clusterProfiler
missMethyl	Bioconductor	https://bioconductor.org/packages/release/bioc/html/missMethyl.html
Survival	R package	https://cran.r-project.org/web/packages/survival/index.html
survMiner	R package	https://cran.r-project.org/web/packages/survminer/index.html
Impute	R package	https://bioconductor.org/packages/release/bioc/html/impute.html
Gviz	Bioconductor	https://bioconductor.org/packages/release/bioc/html/Gviz.html
**Data**		
GDC	NCI	https://portal.gdc.cancer.gov/
cBioPortal	Memorial Sloan	http://www.cbioportal.org/
FireHose	Broad Institute	https://gdac.broadinstitute.org/
Illumina 450k Methylation Annotation	Van Andel Institute	http://zwdzwd.github.io/InfiniumAnnotation

2.2 Supplementary Figures

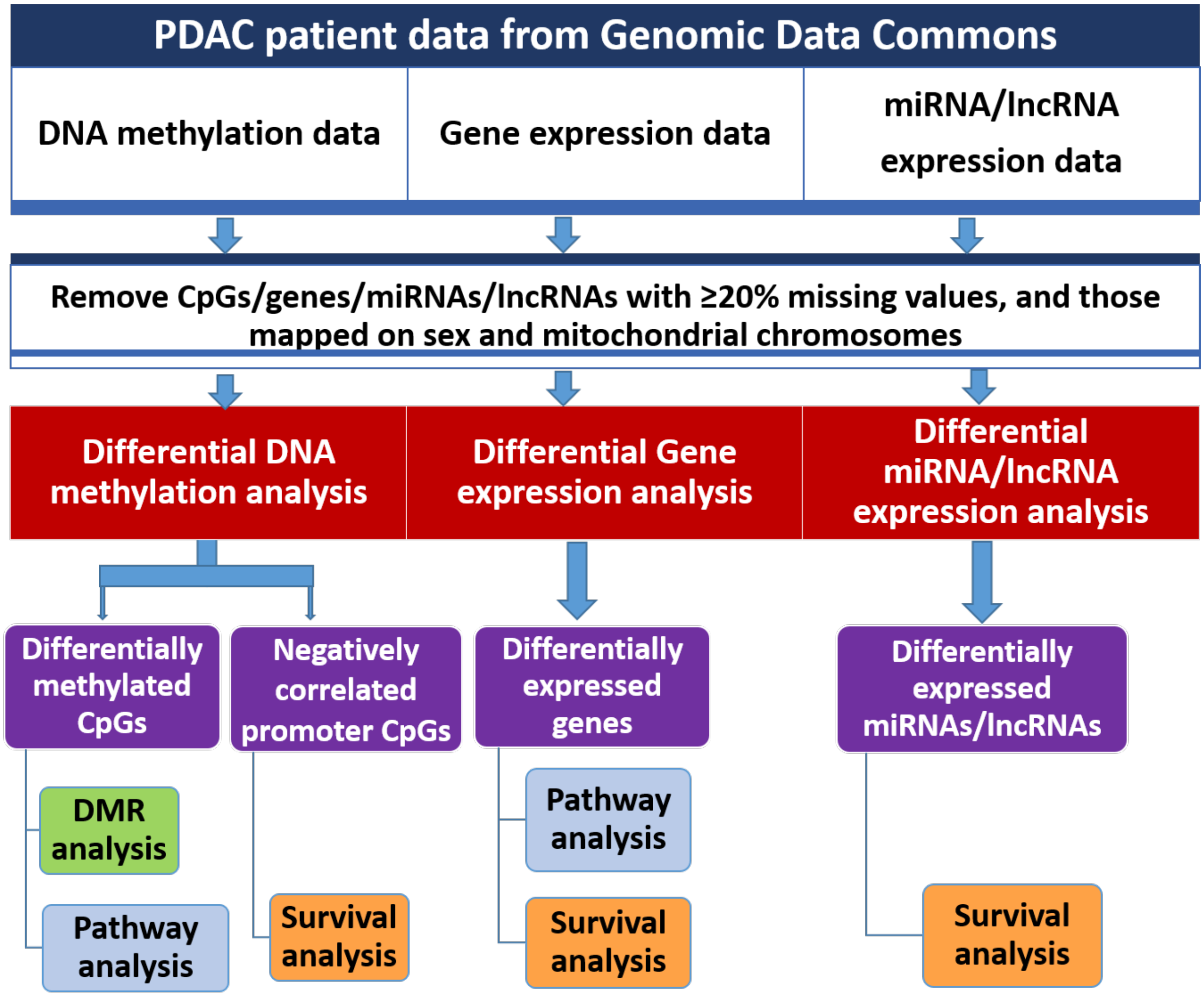

Supplementary Figure 1. Flowchart for the proposed methodology for TCGA pancreatic ductal adenocarcinoma data analysis.

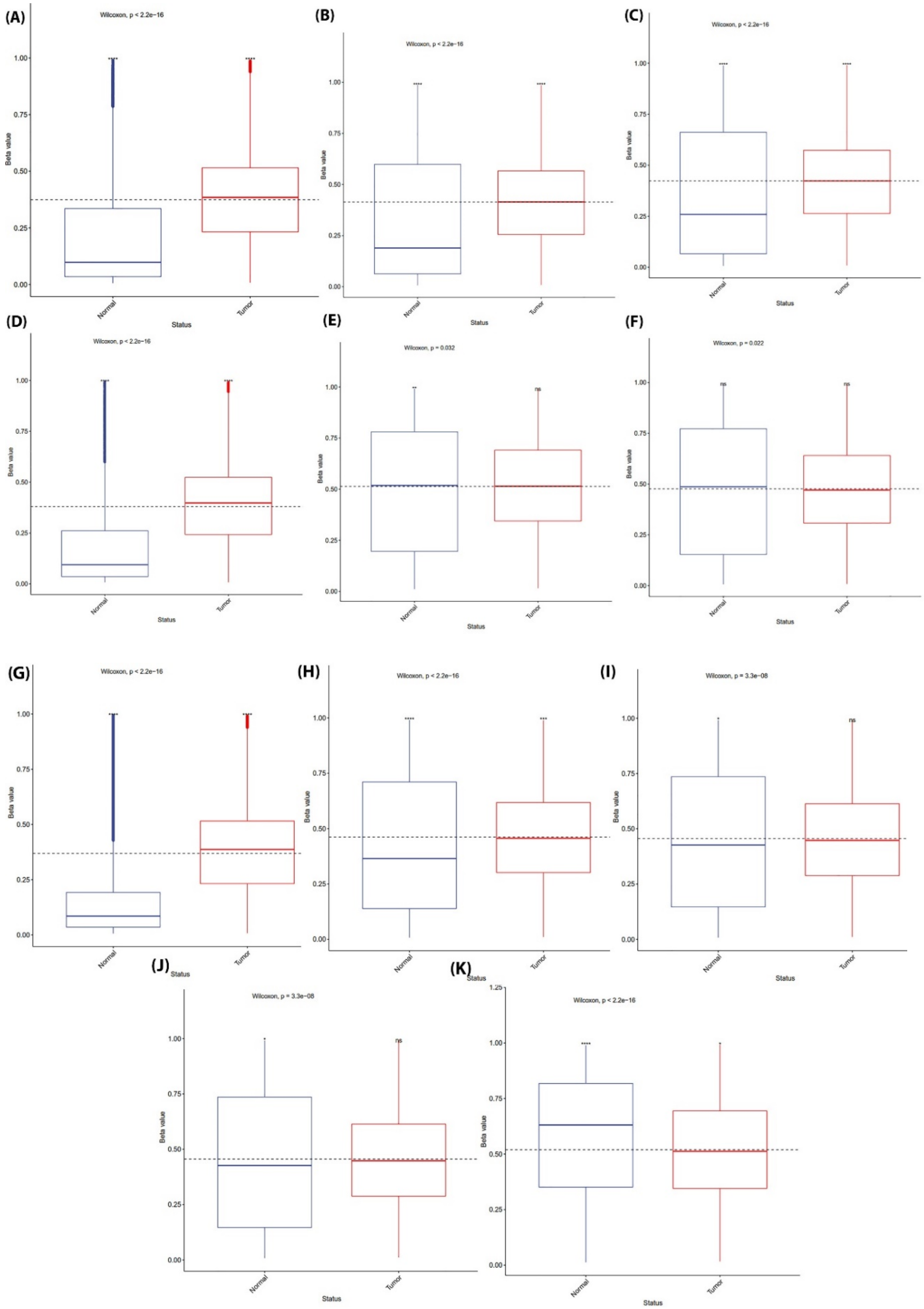

Supplementary Figure 2. DNA methylation distribution of tumor and normal samples in all genomic regions. Boxplot for- (A) TSS200 (B) TSS1500 (C) 5´UTR (D) 1ST Exon (E) Gene body (F) 3´UTR (G) CpG island (H) n-shore (I) s-shore (J) n-shelf (K) s-shelf

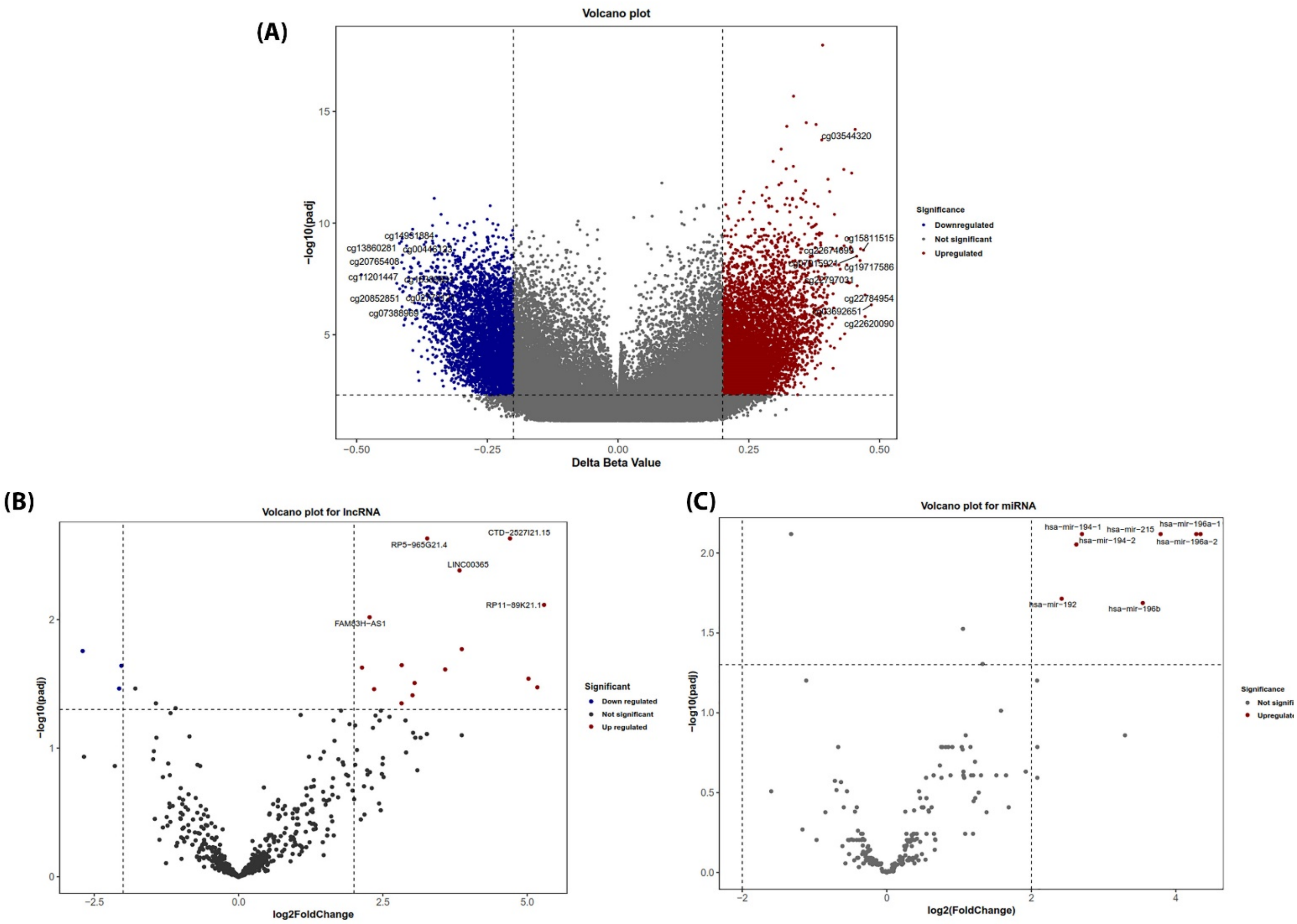

Supplementary Figure 3. Volcano plot for the differentially methylated CpGs, and differentially expressed lncRNA and miRNA. Genes/CpG which are in red and blue colors are highly upregulated and downregulated, respectively in PDAC. Vertical and horizontal dot line represents a cutoff point for log-fold-change p-value respectively

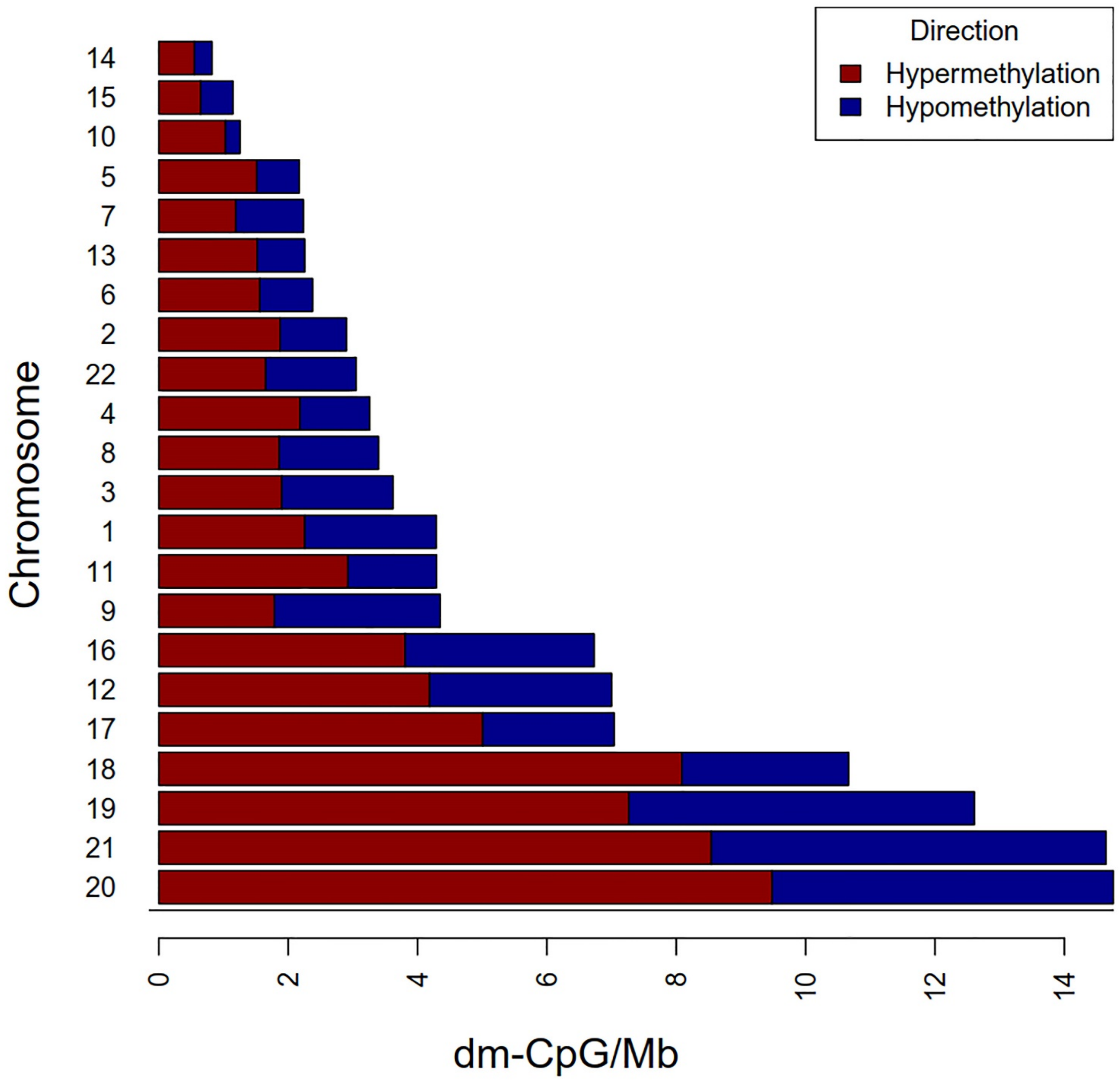

Supplementary Figure 4. Stacked barplot for differential hyper and hypomethylation frequencies for each chromosome. Chromosomes are sorted based on total differential methylation in per megabase pair length of the chromosomes.

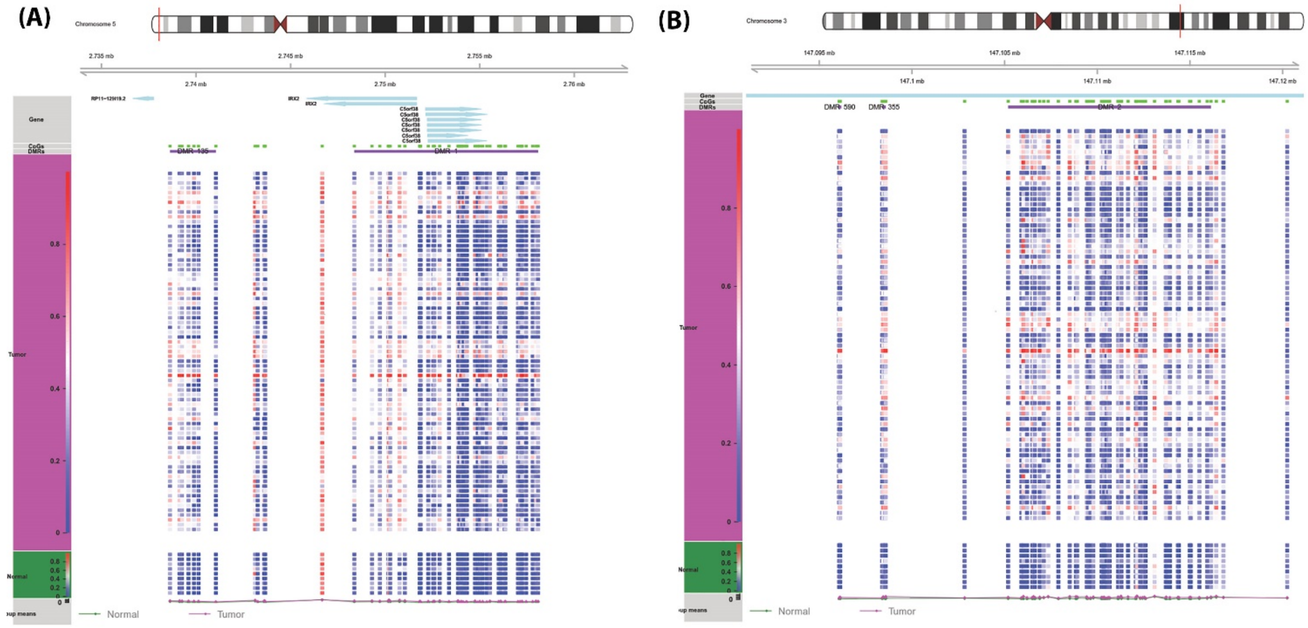

Supplementary Figure 5. *DMRcate* differentially methylated regions (DMRs). (A) DMR1 (B) DMR2

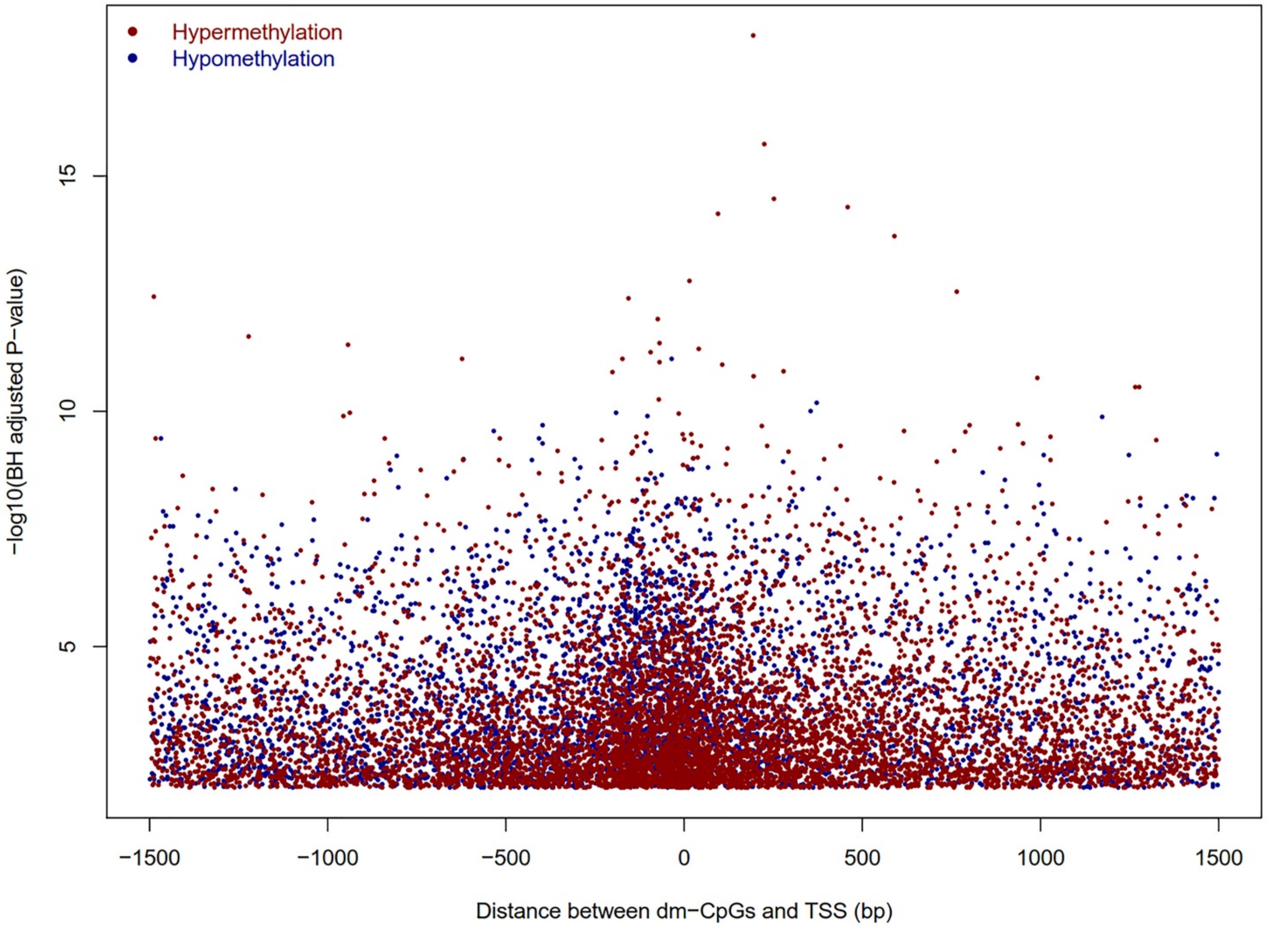

Supplementary Figure 6. CpG sites whose DNA methylation levels were significantly correlated with gene expression with Bonferroni corrected P-value < 0.005
